# Quantitative fluorescence imaging determines the absolute number of locked nucleic acid oligonucleotides needed for suppression of target gene expression

**DOI:** 10.1093/nar/gky1158

**Published:** 2018-11-20

**Authors:** Annette Buntz, Tobias Killian, Daniela Schmid, Heike Seul, Ulrich Brinkmann, Jacob Ravn, Marie Lindholm, Hendrik Knoetgen, Volker Haucke, Olaf Mundigl

**Affiliations:** 1 Roche Innovation Center Munich, Roche Pharma Research and Early Development, Penzberg 82377, Germany; 2 Roche Innovation Center Copenhagen, Roche Pharma Research and Early Development, Hørsholm 2970, Denmark; 3 Roche Innovation Center Basel, Roche Pharma Research and Early Development, Basel 4070, Switzerland; 4 Department of Molecular Pharmacology and Cell Biology, Leibniz-Forschungsinstitut für Molekulare Pharmakologie, Berlin 13125, Germany

## Abstract

Locked nucleic acid based antisense oligonucleotides (LNA-ASOs) can reach their intracellular RNA targets without delivery modules. Functional cellular uptake involves vesicular accumulation followed by translocation to the cytosol and nucleus. However, it is yet unknown how many LNA-ASO molecules need to be delivered to achieve target knock down. Here we show by quantitative fluorescence imaging combined with LNA-ASO microinjection into the cytosol or unassisted uptake that ∼10^5^ molecules produce >50% knock down of their targets, indicating that a substantial amount of LNA-ASO escapes from endosomes. Microinjected LNA-ASOs redistributed within minutes from the cytosol to the nucleus and remained bound to nuclear components. Together with the fact that RNA levels for a given target are several orders of magnitude lower than the amounts of LNA-ASO, our data indicate that only a minor fraction is available for RNase H1 mediated reduction of target RNA. When non-specific binding sites were blocked by co-administration of non-related LNA-ASOs, the amount of target LNA-ASO required was reduced by an order of magnitude. Therefore, dynamic processes within the nucleus appear to influence the distribution and activity of LNA-ASOs and may represent important parameters for improving their efficacy and potency.

## INTRODUCTION

Antisense technologies have experienced significant interest in academia and industry both as research tools and therapeutic agents. As drugs, oligonucleotide based modalities have shown great promise because of their superior target selectivity and potency against otherwise undruggable RNA targets. They can suppress gene expression, modulate mRNA splicing or target non-coding RNAs (ncRNAs) involved in transcriptional and epigenetic regulation (1–5). To reach their intracellular sites of action, oligonucleotides need to overcome cellular membrane barriers such as the plasma membrane and/or the limiting membrane of endosomes (6). Whereas their size and negative charges have long prevented oligonucleotides from crossing lipid membranes (7), introducing chemical modifications has significantly improved their delivery to the cytosol and nucleus. Unassisted uptake of nucleic acid therapeutics has been shown for certain cell types (8,9). The term *gymnosis* (from greek *gymnos*: naked) was coined for locked nucleic acid based antisense oligonucleotides (LNA-ASOs) (10). It refers to an *in vitro* culturing process where unformulated, not further modified or conjugated ‘naked’ LNA-ASOs are taken up with concomitant efficient cytoplasmic or nuclear activity. *Gymnosi*s alleviates the need for transfection reagents and IC50 values are typically found in the micromolar to submicromolar range (10–13).

LNA-ASOs are normally designed as short single stranded 13- to 20-mers containing three structural units: LNA nucleotides, DNA nucleotides and a fully phosphorothioated backbone (14–16). Besides their high resistance to nucleases, they are characterized by their high binding affinity to RNA. Depending on the desired mechanism of action, different oligonucleotide designs have been developed (17,18). In most applications, LNAs are designed as gapmers containing a central DNA nucleotide region flanked at both ends by LNA nucleotides. When hybridized to a complementary RNA target, the DNA/RNA hetero duplex will recruit RNase H1 that cleaves RNA (19). The recruitment of RNase H1 and subsequent RNase H1-mediated cleavage of the target RNA increase the degradation rate of the target RNA by 2- to 4-fold compared with the intrinsic rate of cellular RNA degradation (20,21). The internucleoside phosphorothioate is essential for antisense activity and mediates resistance against nucleolytic degradation. The lipophilic nature of phosphorothioates drives protein binding, binding to cellular membranes and stimulates cellular uptake.

The mechanisms by which LNA oligonucleotides are functionally internalized in cells remain incompletely understood, however there is agreement that multiple endocytotic pathways can be exploited depending on cell type, physiological state or applied LNA-ASO concentration (22–25). The uptake route also appears to affect the activity of internalized oligonucleotides. In primary hepatocytes, functional uptake of unconjugated oligonucleotides has been described to follow a caveolin- and clathrin-independent pathway, which, however, requires the clathrin adaptor AP2 (22), whereas conjugation to *N-*acetyl galactosamine (GalNAc) triggers uptake via classical receptor-mediated endocytosis (26). Following internalization, oligonucleotides traffic through early endosomes, late endosomes and lysosomes with a large fraction being trapped inside late endosomes or lysosomes (23). Apparently, internalized LNA molecules are able to escape from membrane-enclosed vesicles to some extent and reach the cytosol and the nucleus to act on their targets. There is evidence that endosomal release may occur from late endosomes, possibly via membrane fusion processes or conformational changes of the oligonucleotides upon protein binding (27,28).

The subcellular distribution of LNA oligonucleotides has been studied by fluorescence microscopy which allows visualizing fluorescently labelled LNA oligonucleotides inside intact cells. However, due to limited sensitivity, only sites with relatively high accumulation have been detected (22,27–29). Upon unassisted cellular uptake, strong fluorescence signals originating from vesicular structures inside the cells have been observed, whereas functionally relevant LNA-ASO located elsewhere in the cell might have been missed (23).

While RNase H1 is present in mitochondria and in the nucleus (30,31), it is generally assumed that RNase H1 mediated cleavage of mRNA predominantly takes place in the nucleus (32). Delivery into the nucleus is thought to correlate with activity due to nuclear accumulation of fluorescently labelled oligonucleotides after transfection (33,34). The notion of nuclear activity of LNA-ASO is further supported by the fact that antisense oligonucleotides have been shown to efficiently target non-coding RNAs retained in the nucleus (35). However, nuclear LNA-ASO signals have not been detected after gymnotic delivery, which has led to the conclusion that LNA oligonucleotides can efficiently knock down their targets even at very low nuclear concentrations. So far, quantitative information on the cellular distribution of LNA-ASOs (including nuclear content) is not available and the crucial question - how many LNA-ASO molecules need to be present at their site of action - has yet remained unanswered.

In the present study, we have applied quantitative and highly sensitive fluorescence microscopy to measure intracellular LNA-ASO concentrations down to the nanomolar range. The goal of this work was to determine the absolute number of LNA-ASO molecules required for functional knock down of a target RNA. To this end, we delivered a defined amount of LNA-ASO directly into the cytosol via microinjection, thereby circumventing the plasma membrane and endosomal barriers and confirmed the results by gymnotic uptake experiments. The number of injected LNA-ASO molecules was correlated with the degree of functional knock down via quantitative image analysis at the single cell level. In addition, the mobility and diffusion coefficients of nuclear LNA-ASO were measured by fluorescence recovery after photobleaching (FRAP) analysis.

In summary, our studies demonstrate that approximately 10^5^ of the LNA-ASO molecules tested are required to efficiently suppress gene expression of the selected target genes. This was found irrespectively of the route of delivery either by LNA-ASO microinjection into the cytosol or gymnotic LNA-ASO uptake. Following microinjection, LNA-ASOs rapidly translocated into the nucleus where a major fraction appeared immobilized to nuclear components not available for functional knock down. Upon gymnotic uptake of LNA-ASOs via endocytosis a significant amount of LNA-ASOs reached the cytosol/nucleus most likely by endosomal escape. Therefore, improving targeted accumulation at desired tissues/cells and dynamic processes within the nucleus represent important parameters for extending the therapeutic applications of LNA-ASOs.

## MATERIALS AND METHODS

### Oligonuncleotides and antibodies

**Table:** 

MALAT1:	GAGttacttgccaACT
MALAT1-AF488:	GAGttacttgccaACT-AF488-3′
HIF1A:	GCaagcatcctGT
FAM-HIF1A:	5′-FAMS1GCaagcatcctGT
AF647-HIF1A:	5′-AF647-GCaagcatcctGT
AF594-HIF1A:	5′-AF594-GCaagcatcctGT
Atto647N-HIF1A:	5′-Atto647N-GCaagcatcctGT
Unrelated:	CcAAAtcttataataACtAC

The MALAT1 ASO sequence was previously published (36), the HIF1A sequence was published in the patent US20100249219A1, SEQ ID NO: 14. Single-stranded LNA oligonucleotides were synthesized using standard phosphoramidite chemistry. Upper case denotes LNA, lower case DNA. All sequences are full phosphorthioate. DNA phosphoramidites and Atto647N N-hydroxy succinimidyl (NHS) ester were purchased from Sigma-Aldrich (St. Louis, MO), and LNA phosphoramidites were produced in house (LNA phosphoramidites are also commercially available from QIAGEN [Hilden, Germany]). 5′-aminolinker C6 and FAM phosphoramidite was purchased from Link Technologies (Bellshill, Scotland). 3′-aminolinker C6 CPG was purchased from Chemgenes (Wilmington, MA, USA). Alexa Fluor (AF488, AF594 and AF647) *N*-hydroxy succinimidyl (NHS) esters were purchased from Fisher Scientific (Slangerup, Denmark)

Unconjugated and amino linker oligonucleotides were synthesized on NittoPhase HL UnyLinker 350 support (Kinovate, Oceanside, CA, USA) on a MerMade 12 synthesizer (Bioautomation, Irving TXmark) at 20 μmol scale. After synthesis, the oligonucleotides were cleaved from the support using aqueous ammonia at 65°C overnight. The oligonucleotides were purified by ion exchange on SuperQ-5PW gel (Tosoh Bioscience, Griesheim, Germany) and desalted using a Millipore membrane. After lyophilization, the oligonucleotide was characterized by LC–MS (reverse phase and electrospray ionization-mass spectrometry).

Alexa Fluor (AF488, AF594 and AF647) and Atto647N labelled oligonucleotides were synthesized by conjugation of 4 equivalents of the corresponding Alexa Fluor *N*-hydroxy succinimidyl (NHS) ester with a 5′- or 3′-aminolinker oligonucleotide in 20 mM aqueous sodium hydrogen carbonate. After 5–16 h, the reaction mixture was applied directly to reversed-phase HPLC purification (XBridge Peptide BEH C18 OBD Prep, 300A, 10 μm, 10 × 150 mm column and 0.1M ammonium acetate and acetonitrile as eluent). Pooled fractions were lyophilized and precipitated from 300mM sodium acetate and ethanol to obtain the sodium salt. The oligonucleotide was characterized by LC–MS.

Primary antibody: HIF1A (Clone 54, 610958, BD Bioscience). Secondary antibody: anti-mouse-AF594 (115-585-164, Jackson)

### Cell culture

MCF-7 cells (NCI) were grown in Roswell Park Memorial Institute (RPMI) medium supplemented with 10% fetal calf serum (FCS) and 100 U/ml penicillin and 100 μg/ml streptomycin. The cell line was verified as pathogen-free and identity was verified by STR-PCR analysis before use. Cells were subcultured every 2–3 days and incubated at 37°C in a humidified atmosphere containing 5% CO_2_.

### Microinjection

For microinjection, MCF-7 cells were either seeded onto 35-mm μ-dishes with glass bottom grid (μ-ibidi, Martinsried, Germany) or 35-mm WillCo dishes (WillCo Wells B.V., Amsterdam, Netherlands) containing glass coverslips with grid (Celllocate, Eppendorf, Hamburg, Germany). Glass surfaces had been coated with 30 μg/ml fibronectin in PBS for 1 h at 37°C. Microinjection was performed using a FemtoJet Microinjector (Eppendorf, Hamburg, Germany) with sterile Femtotips^®^ I capillaries with a 0.5 μm inner diameter and 1 μm outer diameter. Injected material was diluted in injection buffer containing 48 mM K_2_HPO_4_; 4.5 mM KH_2_PO_4_; 14 mM NaH_2_PO_4_; pH 7.2.The following injection parameters were applied: Injection pressure pi: 120 hP, compensation pressure pc: 5 hP, injection time ti: 0.2 s.

### Calibration of microinjection via photon counting imaging

In order to calibrate the number of molecules injected into the cell, intracellular concentrations of tracer molecules after microinjection were measured by photon counting imaging. One day prior to the experiment 1 × 10^5^ MCF-7 cells were seeded into 35-mm μ-dishes with glass bottom grid (ibidi, Martinsried, Germany) which had been coated with 30 μg/ml fibronectin. On the next day, cells were injected with 10 kDa, anionic, fixable dextran-AF488 and dextran-AF647 (ThermoFisher) using the injection parameters described above. Injection buffer containing 5 μM dextran-AF647 + 1 μM/0.5 μM/0.1 μM dextran-AF488 was centrifuged at 13 200 g for 3 min and sonicated before injection. Concentrations of injection solutions were confirmed by absorption measurements using a Nanodrop spectrophotometer based on the published molar extinction coefficient (ϵ) for Alexa Fluor 488 (73 000 cm^−1^ M^−1^; www.aatbio.com/resources/extinction-coefficient/Alexa_Fluor_488). Conjugation to dextran did not measurably affect the spectral properties of the AlexaFluor 488 dye as confirmed by excitation and emission spectra. For every experimental condition, 10 injected cells were analyzed on a Leica SP5X confocal microscope directly after injection. Intracellular tracer concentrations were measured using hybrid detectors (HyD) in photon counting mode. Imaging conditions were as follows: 63×/1.2 NA water immersion lens, white light laser excitation at 488 and 647 nm, emission band pass at 495–550 and 656–758 nm, pinhole AU = 1.0, pixel size 72.9 nm and 8-bit resolution. For quantification, solutions with defined concentrations of dextran-AF488 and dextran-AF647 were measured and a calibration curve was established. For every image pixel, photon counts were translated into absolute concentrations of dextran-AF488 and dextran-AF647, respectively. Image segmentation was carried out to identify injected cells using an analysis pipeline built in CellProfiler.

### Calibration of microinjection via fluorescence correlation spectroscopy

In parallel to photon counting imaging, intracellular tracer concentrations were measured using fluorescence correlation spectroscopy (FCS) within the same cells. For FCS, temporal intensity fluctuations within the confocal volume were recorded and concentrations were calculated from the amplitude of the autocorrelation of the time-resolved signal. FCS measurements were carried out on a Leica SP5X confocal microscope equipped with external APD detectors and TCSPC electronics (PicoHarp300, PicoQuant, Berlin) at 37°C. As excitation source, a pulsed white light laser with a repetition rate of 80 MHz was used at emission wavelengths set to 488 and 633 nm.

The light was focused onto the sample via a 63×/1.2 NA water immersion objective lens and the resulting fluorescence was collected through the same objective. Emission light originating from AF488 and AF647 were divided via a HC BS 560 beamsplitter (Semrock), separated from the laser light by 535/70 ET bandpass and 685/70 ET bandpass filters (both from Chroma), and focused onto an avalanche photodiode detector (MPD micro photon devices, PicoQuant) operated in single photon counting mode. All measurements and data analysis were performed using the SymPhoTime software integrated into the FCS wizard of the Leica LAS acquisition software. Guided by the FCS wizards, the following acquisition steps were carried out: First, the motorized correction collar was adjusted to correct for the thickness of the glass surface of the chamber slide. The excitation laser power was adjusted such that the dye was not oversaturated which would lead to an overestimation of the confocal volume. To perform point measurements inside the injected cells, single cells were centered above the objective and imaged by scanning in the xz-dimension. Imaging scans allowed positioning of the confocal volume at locations suitable for FCS measurements. Intensity fluctuations within the confocal volume were recorded for 30 sec on every spot and three spots were analyzed per cell. Autocorrelation curves were calculated from the recorded time traces and fit to a 2D-diffusive model with a triplet term assuming a triplet lifetime of 4 μs (37). The average number of molecules present in the confocal volume was obtained from the fit and translated into concentrations by calibrating the confocal volume (38). Solutions of Atto488-NHS and AF647-NHS were routinely used to calibrate the instrument prior to measurements. Knowing the diffusion coefficient of both dyes, the effective volume was determined analytically from the FCS fit of the samples (400 and 330 μm²/s, respectively (39).

### Immunofluorescence and FISH

For detection of MALAT1 RNA fluorescence *in situ* hybridization (FISH) was performed using Stellaris FISH probes according to the manufacturer's protocol (LGC Biosearch, Steinach, Germany). In brief, treated cells were fixed with 4% *para*-formaldehyde (PFA) for 20 min, washed two times with PBS and incubated for at least 4 h in 70% ethanol at 4°C. Samples were stored in ethanol at 4°C for up to 1 week. After one washing step with the provided washing buffer A, samples were incubated over night at 37°C in a humidified chamber with hybridization buffer containing 12.5 nM Quasar®570-labelled probes (MALAT1: SMF-2035-1, GAPDH: SMF-2026-1) and dimethylformamide. Samples were then washed with washing buffer A for 30 min at 37°C, and nuclei were stained with DAPI (5 ng/ml in washing buffer A) for 30 min at 37°C. Subsequently, samples were washed for 5 min with washing buffer B and mounted using Vectashield mounting medium (Vectorlabs, Burlingame, CA, USA). Samples were allowed to dry for 1 h and imaged on the same day.

For detection of proteins by immunofluorescence, samples were prepared as described (40). In brief, PFA-fixed samples were washed with PBS with increasing salt concentration and incubated for 30 min with goat serum dilution buffer (GSDB; 0.45 M NaCl, 20 mM phosphate buffer, 0.3% Triton X-100, 17% goat serum) in order to permeabilize membranes and block nonspecific antibody binding sites. Cells were next incubated for 1 h with primary antibodies prepared in GSDB (HIF1A: 5 μg/ml). Following three washes with high-salt PBS, cells were incubated for 30 to 90 min with fluorescent secondary antibodies prepared in GSDB (1:100). Cells were washed with PBS with decreasing salt concentration and nuclei were counterstained with DAPI at 1 μg/ml for 5 min before mounting the coverslips onto glass slides using freshly prepared mounting solution containing 70% glycerol in PBS.

### Quantitative analysis of target gene knock down in single cells

Single cell analysis of target knock down was carried out on a Leica SP5X confocal microscope using a 40×/1.25 NA oil immersion objective lens. Injected cells were located with the help of the Celllocate grid on the glass cover slide. Sequential scans were performed using white light laser excitation at 405 nm, 488 nm, 561 nm or 594 nm. Fluorescence emission was detected at 415–480 nm (DAPI), 495–530 nm (AF488), 550–560 nm (Quasar^®^ 570) or 600–700 nm (AF594) using HyD detectors. Image format: 512 × 512 pixel, image size: 193.75 × 193.75 μm². Acquisition parameters were kept constant within the experiment to ensure comparable signal levels. Images were segmented and nuclear mean fluorescence intensities (grey values) were calculated using an automated image analysis pipeline implemented in Cellprofiler. Nuclei were identified using the DAPI channel, estimating a typical object diameter of 20–150 pixel and discarding objects outside the diameter range. Three-classes thresholding was performed using the Otsu method minimizing the weighted variance. The middle intensity class was assigned to background. According to their shape, touching objects were automatically separated using dividing lines. Nuclear mean intensities of tracer and target signals were calculated as percentage of maximal intensity (255 grey values). Target vs tracer intensity was visualized on a scatter plot to manually define a tracer intensity threshold for identification of injected cells. Statistical analysis was performed using GraphPad Prism (La Jolla, CA, USA).

### Live cell imaging of nuclear LNA-ASO accumulation

MCF-7 cells were grown in 35-mm μ-dishes with glass bottom grid (μ-ibidi, Martinsried, Germany) to 50–70% confluency and co-injected either with 5 μM dextran-AF488 (10 kDa) + 5 μM AF647-HIF1A LNA or 5 μM dextran-TMR (70kDa) + 5 μM AF488-MALAT1 LNA-ASO as described above. Immediately after injection, cells were transferred to a Leica SP8X confocal microscope equipped with a stage-top incubator to maintain temperature, CO_2_ and humidity (Oko-touch, Okolab, Ottaviano, Italy). Acquisition of time series was started approximately two minutes after injection using a 63×/1.2 NA water immersion objective lens and HyD detectors under adaptive focus control with a frame interval of 10 s. For ATP depletion, cells were transferred to starvation conditions over night (DMEM with 1% FCS and 0.1% glucose, PAN Biotech, Cat.No. P04-03556). 45 min prior to injection, cells were incubated with 6 mM deoxyglucose and 10 mM sodium azide in phenolred-free medium and kept therein during injection and image acquisition.

### Staining of RNA via click-chemistry

Freshly synthesized RNA was labelled using the Click-iT™ RNA Alexa Fluor™ 594 Imaging Kit (ThermoFisher) as described by the manufacturer. In brief, MCF-7 cells were incubated with 1 mM 5-ethynyl uridine (EU) for 1 h. After fixation with 4% PFA for 20 min, cells were washed once with PBS and permeabilized by incubating for 15 min with GSDB. After washing with PBS, cells were incubated 30 min at room temperature with the Click-iT™ reaction cocktail containing CuSO_4_ and AF594-azide. Subsequently, cells were washed twice with PBS before mounting the coverslips onto glass slides using ProLong™ Gold Antifade Mountant (ThermoFisher).

### Fluorescence recovery after photobleaching

In order to measure intracellular diffusion coefficients of LNA, fluorescence recovery after photo bleaching (FRAP) experiments were performed with microinjected cells. To this end, MCF-7 cells were injected with 20 μM stock FAM-LNA, incubated for 30 min at 37°C and 5% CO_2_ and analyzed on Leica SP5X confocal microscope using a 63×/1.2 NA water immersion objective lens. FRAP experiments were conducted using the in-built FRAP wizard. An Argon laser set to maximum intensity (λ_emission_: 488 nm) was used to bleach the fluorophore within a circular region of interest with 4 μm diameter (1 frame, 97 ms) and the recovery of the fluorescence intensity within the bleached area was recorded over time (70 frames, 97 ms). Ten frames were acquired before photobleaching for the region of interest (ROI). The following imaging parameters were chosen for fast acquisition of the recovery: Scan speed: 1400 Hz, bidirectional scan, format: 256 × 256 pixel, image size: 30.75 × 30.75 μm2, AOTF setting: 5%, emission detection range: 496–600 nm. Fluorescence intensities within the bleached area (ROI1), an unbleached area within the nucleus (ROI2) and background area (ROI3) were measured using the ROI manager tool implemented in ImageJ (NIH, Bethesda, MD, USA). Recovery curves were background subtracted, corrected for photobleaching and normalized either to prebleach intensities (for representation of immobile/mobile fractions) or to postbleach intensity at infinity (for calculation of diffusion coefficients). In the latter case, normalized recovery curves were fitted to an exponential model: *F*(*t*) = *a*(1 – exp(1/τ)) + *c* using the Matlab software environment (Mathworks, MA, USA). Diffusion coefficients were calculated from the half-life of the recovery using the following relationship: *D* = 0.88*r*²/4*t*_1/2_ with *t*_1/2_ = τ ln(2).

FRAP measurements in solution were carried out in 8-well chamber slides (Nunc™ Lab-Tek™, ThermoFisher) using 5 μM solutions of FAM-HIF1A LNA-ASO at 20 μm distance from the glass surface. Acquisition parameters were the same as for cellular measurements except that the diameter of the bleached area was adjusted to 10 μm to account for faster recovery kinetics.

### Gymnotic cultures

MCF-7 cells (7500 cells/well) were seeded into 8-well chamber slides (Nunc™ Lab-Tek™, ThermoFisher) and allowed to adhere overnight. Cells were incubated with AF594-HIF1A-LNA-ASO for 24 h/48 h/72 h and a heat map of intracellular LNA-ASO concentrations was generated by photon counting imaging of living cells. Therefore, cells were washed three times with PBS and imaged in phenolred-free medium using a Leica SP5X confocal microscope equipped with a humidified and temperature controlled stage-top incubator (INU, Tokai hit, Fujinomiya, Japan). Intracellular tracer concentrations were determined using hybrid detectors (HyD) in photon counting mode. Imaging conditions were as follows: 63×/1.2 NA water immersion lens, white light laser excitation at 594 nm, emission band pass at 600–700 nm, pinhole AU = 2.0, pixel size 300 nm, scan speed 400 lines/s and 12-bit resolution. 10–15 image frames were accumulated to collect enough photons from dim cellular structures. This acquisition procedure allowed displaying the whole dynamic range including bright vesicular and dim nuclear signals. For quantification, a dilution series of LNA-AF594 ranging from 0.1 to 1 μM were measured using the same imaging conditions to establish a calibration curve. Photon counts were then translated into concentrations and displayed as pseudo colour intensity heat map using a logarithmic colour bar such that differences in the low concentration range could be visualized. To quantify nuclear and vesicular concentrations, vesicles were detected using an analysis pipeline built in CellProfiler whereas nuclei were identified manually using transmission images such that signal contamination originating from vesicular structures was prevented. The number of vesicles/cell was determined from maximum projections of 3D volumes of 328 analyzed cells using the image analysis pipeline implemented in CellProfiler for 2D images.

### Knock down analysis-qPCR

Cells were seeded in 96-well plates at a density of 3500 cells/well and allowed to adhere overnight. LNA-ASO solutions were diluted in phosphate buffered saline and added to the cells at the indicated concentrations. After 72 h of LNA-ASO exposure, medium was removed and gene expression levels were analyzed by RNA isolation and real-time qPCR. Total RNA was isolated with the PureLink™ Pro 96 total RNA Purification Kit (Thermo Fisher Scientific; Waltham, MA, USA) according to the manufacturer's protocol. Real-time PCRs were prepared as 10 μl reactions containing 10-fold diluted total RNA, qScript XLT 1-Step RT-qPCR ToughMix (Quantabio; Beverly, MA, USA) and TaqMan gene expression assays (Assay ID HIF1A: Hs00936368; Assay ID MALAT1: 00273907; Assay ID GAPDH control: Hs99999905; Thermo Fisher Scientific; Waltham, MA, USA). 1-Step RT-qPCR was performed in 386-well plates with a LightCycler 480 II (Roche Molecular Systems; Pleasanton, CA). Target and reference gene expression were analyzed in the same reaction by multiplex PCR with a FAM labelled probe for the target gene and a VIC labelled probe for the reference gene. Relative gene expression levels were determined by the standard curve method. PCRs were performed in duplicates.

## RESULTS

### Quantification of the number of microinjected LNA-ASO molecules required for gene knock down

**Figure 1.**
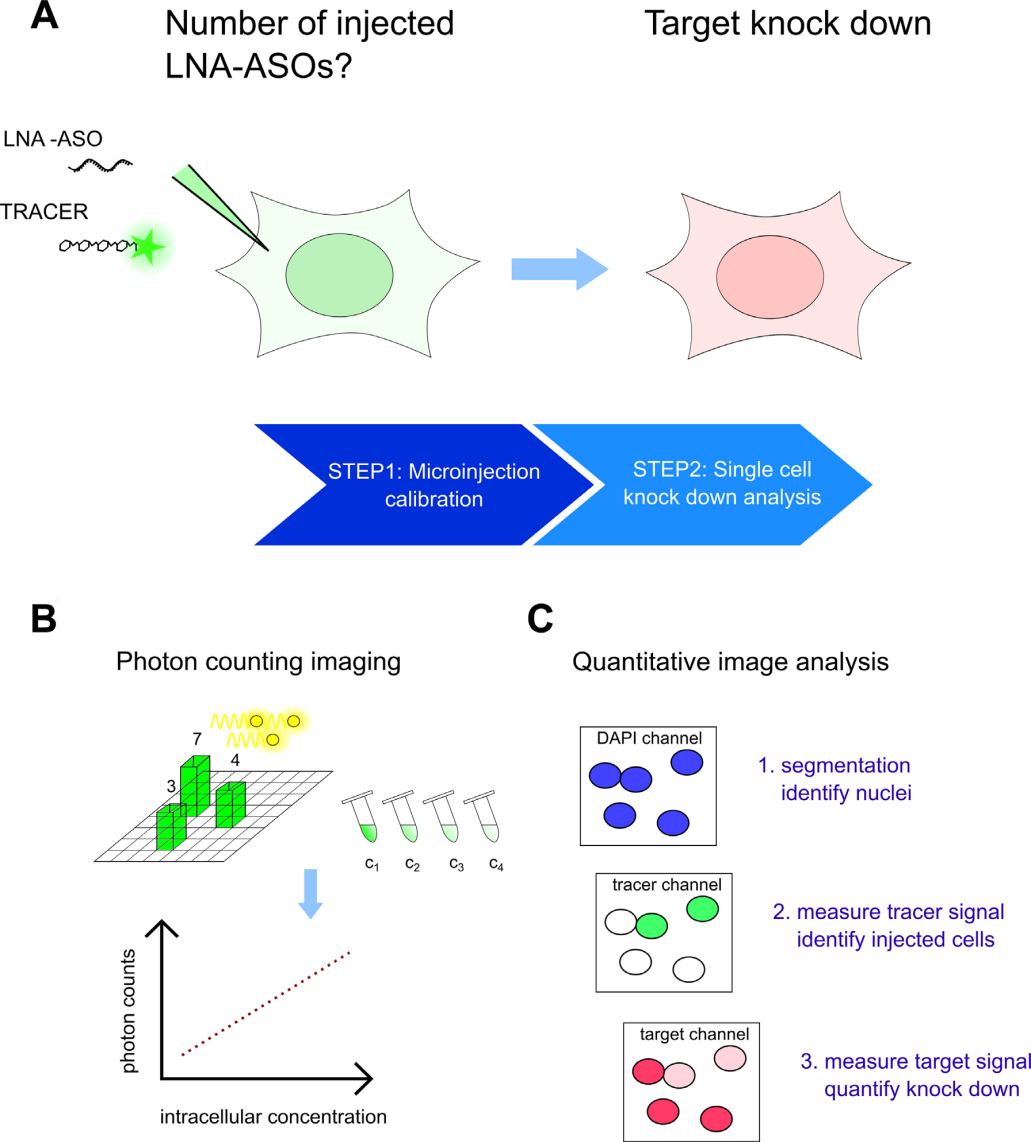
Delivery of LNA-ASOs by microinjection and single cell knock down analysis. The number of LNA-ASO molecules required for target knock down was determined by delivering a defined amount of LNA-ASOs into the cytosol via microinjection and subsequent single cell knock down analysis in injected cells (**A**). Calibration experiments were performed to determine the number of injected molecules. The fluorescence signal of a tracer molecule was used to measure intracellular concentrations via fluorescence correlation spectroscopy (FCS) and photon counting imaging (**B**). Target knock down was detected in injected cells on RNA and protein level by fluorescence *in-situ* hybridization and immunofluorescence, respectively. RNA and protein levels were assessed by quantitative image analysis. Using an automated analysis pipeline, cell nuclei were first identified by image segmentation. Thereafter, mean fluorescence intensities of tracer and target signals in the segmented cell nuclei were calculated. Injected cells were distinguished from non-injected cells using the tracer signal (**C**).

To address the question how many LNA-ASO molecules are required for effective gene knock down we followed first a single-cell analysis approach, combining microinjection of LNA-ASO with quantitative confocal fluorescence microscopy (Figure 1A). Microinjection enables the direct delivery of LNA-ASO molecules into the cytosol, thereby bypassing the cellular membrane and uptake into endosomes and avoiding transfection-based reagents that may compromise membrane integrity.

#### Calibration

We calibrated the experimental setup by delivering a defined amount of LNA-ASO directly into the cytosol via microinjection. We then analyzed the efficacy of target knock down in the injected cells either on the RNA or on the protein levels via quantitative fluorescence imaging of single cells. The amount of LNA-ASO injected into the cells cannot specifically be calculated, as the injection volume depends on both, the applied injection pressure and on time. To measure the actual injection volume, we determined the concentrations of labelled molecules inside living cells by quantitative confocal imaging using HyD detectors set to photon counting mode, which directly translates into the effectively delivered amount of substance (Figure 1B). Fluorescently labelled dextran molecules were microinjected into the cells at different concentrations ranging from 100 nM to 5 μM (Supplementary Figure S1). Intracellular concentrations obtained from confocal imaging in photon counting mode were validated by fluorescence correlation spectroscopy (FCS), a technique that measures intensity fluctuations within the focal volume depending on the fluorophore concentration (Supplementary Figure S2) (41). We were able to detect fluorescently labelled dextrans at intracellular concentrations down to 1 nM corresponding to roughly 1/10 (0.079 ± 0.008, mean ± SD) of the injected concentrations. For example, injection of a 1 μM stock solution resulted in an average intracellular concentration of ∼100 nM (114 ± 36 nM, mean ± SD). Assuming an average cellular volume of 2000 fl (42), the injected volume was roughly 200 fl containing ∼10^5^ (1.37 ± 0.43 × 10^5^, mean ± SD) molecules. These data verified that quantitative confocal fluorescence microscopy at the single cell level allows determining the total number of injected fluorescent molecules.

#### Knock down analysis at RNA level

Having successfully calibrated the conditions for microinjection, we determined the efficiency of target RNA knock down following delivery of a defined amount of LNA-ASO into the cells. Long non-coding MALAT1 RNA was used as a model as this RNA is retained within the nucleus (43). Knock down of RNA within injected cells was detected by fluorescence *in situ* hybridization using a mixture of fluorescently labelled MALAT1 probes designed to specifically hybridize to complementary regions of the MALAT1 RNA sequence (Supplementary Figure S3). We verified that the presence of LNA-ASO did not interfere with the target hybridization of the probe (Supplementary Figure S4). To analyze the time course of target RNA suppression, MALAT1 RNA levels were determined at different time points after co-injection of defined concentrations of unlabelled MALAT1 LNA-ASO and a fluorescent tracer (Supplementary Figure S5). Already 2 h post-injection the MALAT1 RNA signal was clearly reduced in the injected cells. Knock down of the target RNA persisted to 24 h, the latest observed time point. Based on these data we decided to analyze knock down efficiencies 4 h post-injection in all subsequent experiments.

**Figure 2.**
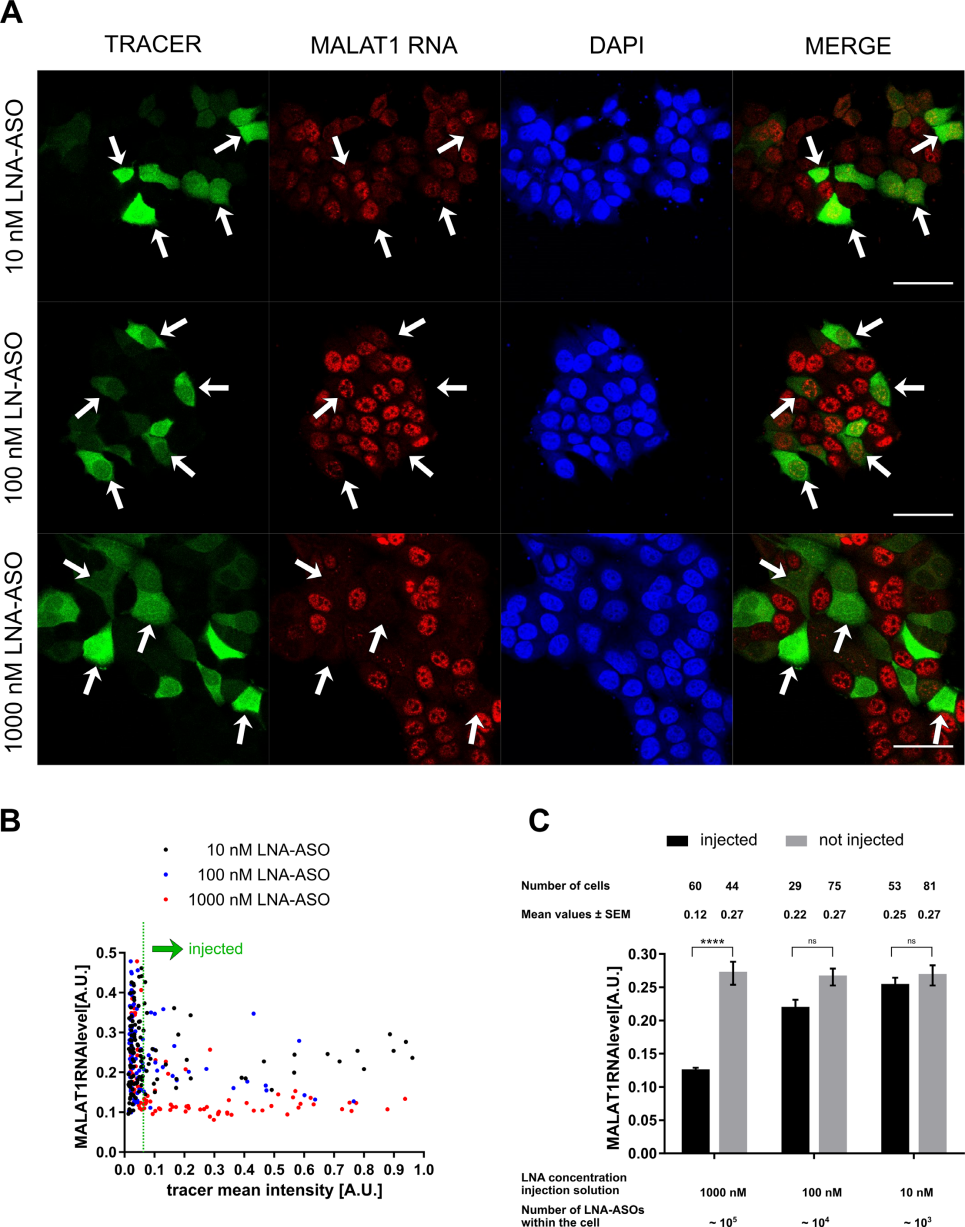
MALAT1 RNA knock down in microinjected cells. MCF-7 cells were microinjected with a solution of 10 μM dextran-AF488 as tracer + 1000 nM/100 nM/10 nM unlabelled MALAT1 LNA-ASOs. Cells were incubated for 4 h, and fixed with 4% PFA. MALAT1 RNA was detected via fluorescence *in situ* hybridization. Fluorescence microscopy revealed knock down of MALAT1 RNA in injected cells at high LNA-ASO concentrations. White arrows indicate injected cells. Scale bars: 50 μm (**A**). The cellular fluorescence signal originating from MALAT1 RNA staining was quantified. Injected and non-injected cells were distinguished by defining a threshold of the tracer signal (**B**). Intracellular concentrations of LNA-ASOs after microinjection were estimated from calibration experiments. Mean values ± standard error of the mean (SEM) of cellular MALAT1 RNA levels are depicted. 20 000–200 000 LNA-ASO molecules need to be injected into the cells to observe significant knock down of MALAT1 RNA. Statistical significance was assessed with a two-way ANOVA and Tukey posttest. The degree of significance is ****P < 0.0001.

**Figure 3.**
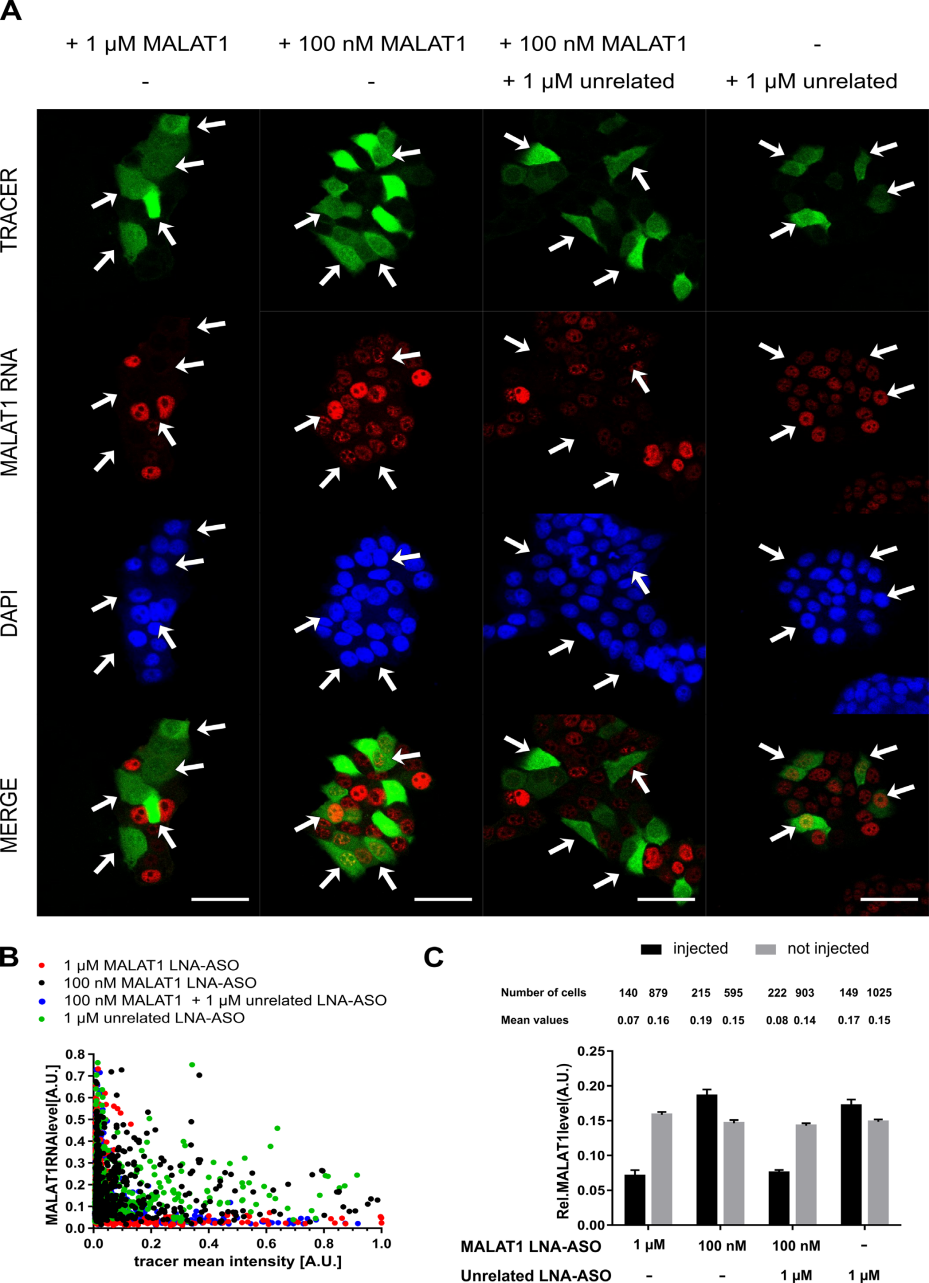
Competition experiment using unrelated LNA-ASOs. MCF-7 cells were microinjected with a solution of 10 μM dextran-AF488 as tracer + target LNA-ASOs (MALAT1) + unrelated LNA-ASOs at the indicated concentrations. After microinjection, cells were incubated for 4 h, and fixed with 4% PFA. MALAT1 RNA was detected via fluorescence *in situ* hybridization. White arrows indicate injected cells. Scale bars: 50 μm (**A**). Knock down of target RNA was assessed by quantitative image analysis. Scatter plot representation of relative MALAT1 RNA levels versus relative tracer signals (**B**). Mean values ± standard error of the mean (SEM) of cellular MALAT1 RNA levels are depicted (**C**).

To determine the number of LNA-ASO molecules required for efficient suppression of MALAT1 RNA, the LNA-ASO injection concentration was adjusted from 1 μM to 10 nM (Figure 2). The MALAT1 RNA signal was virtually eliminated in all cells injected with a 1 μM LNA-ASO solution (corresponding to ∼10^5^ intracellular LNA-ASO molecules). In contrast, MALAT1 RNA signals remained detectable in cells injected with lower LNA-ASO concentrations and in all non-injected cells (Figure 2A). To determine the efficacy of knock down quantitatively, we injected a large number of cells and performed automated image analysis to measure RNA levels in injected vs non-injected cells (Figure 1C): Using the DAPI channel, cell nuclei were identified via image segmentation. Subsequently, nuclear mean fluorescence intensities of the tracer and the target RNA signals were determined. Injected cells were distinguished from non-injected cells by defining a threshold based on the intensity of the tracer. Statistical analysis revealed that RNA levels were significantly reduced by more than 50% in cells treated with an average number of ∼10^5^ LNA-ASO molecules (Figure 2B). An average amount of ∼10^4^ LNA-ASO molecules resulted in a minor reduction of MALAT1 RNA levels, whereas ∼10^3^ LNA-ASO molecules did not yield any measurable knock down. Microinjection of non-targeting LNA-ASO into the cytoplasm had no effect on target RNA levels (Figure 3). In a competition experiment in which the target LNA-ASO was co-injected together with an excess of unrelated LNA-ASO, the presence of unrelated non-targeting LNA-ASO significantly decreased the number of target LNA oligonucleotides required for knock down to 10^4^ molecules (Figure 3).

#### Knock down analysis at protein level

**Figure 4.**
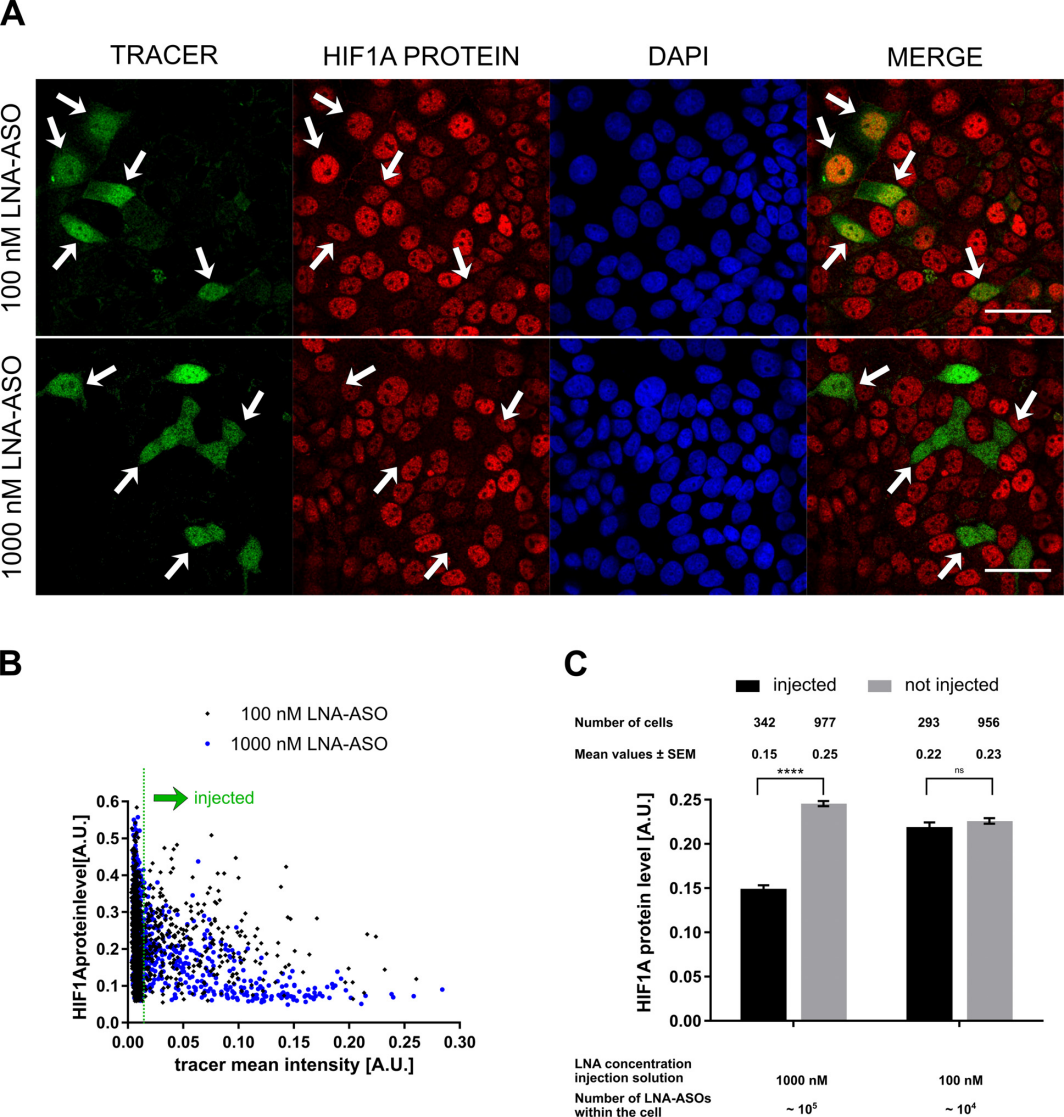
HIF1A protein knock down in microinjected cell. MCF-7 cells were microinjected with a solution of 10 μM dextran-AF488 as tracer + 1/0.1 μM unlabelled HIF1A LNA-ASOs. Cells were incubated with 100 μM deferoxamine for 48 h, and fixed with 4% PFA. HIF1A protein was detected via HIF1A-antibody immunocytochemistry. Fluorescence microscopy revealed knock down of HIF1A protein in injected cells at high LNA-ASO concentrations. White arrows indicate injected cells. Scale bars: 50 μm (**A**). Knock down analysis in injected cells was performed using quantitative image analysis. The cellular fluorescence signal originating from HIF1A protein staining was quantified. Injected cells were distinguished from non-injected cells using the tracer signal (**B**). Mean values ± standard error of the mean (SEM) of cellular HIF1A protein levels in injected and non-injected cells are depicted. Statistical significance was assessed with a two-way ANOVA and Tukey posttest. The degree of significance is ****P < 0.0001 (**C**).

Proteins are, on average, about 2,800 times more abundant and five times more stable than their corresponding transcripts (44). The time course of protein knock down is more complex as it is determined as a function of transcription and translation rate and protein half-life. Depending on those parameters, depletion of target proteins is usually detected at much later time points compared to RNA (10,45). To determine the number of LNA-ASO molecules required for protein knock down we analyzed the levels of the short-lived transcription factor HIF1A. While HIF1A mRNA is constitutively expressed and transcribed, HIF1A protein exhibits a half-life of less than 5 min in the presence of oxygen (46), but is stabilized under conditions of hypoxia. Indeed, treatment of cells with 100 μM deferoxamine, simulating hypoxic conditions, resulted in bright nuclear HIF1A protein staining after 48 h incubation (Figure 4A). Deferoxamine-treated cells were co-injected with 1 μM or 0.1 μM unlabelled LNA-ASO targeting HIF1A and 10 μM of dextran-AF488 as a fluorescent tracer. Injection of 1 μM LNA-ASO led to a substantial reduction of HIF1A immunoreactivity, while injection of 0.1 μM LNA-ASO was insufficient (Figure 4A+B). Statistical analysis of several hundred injected cells confirmed that ∼10^5^ LNA-ASO molecules were required to induce functional HIF1A protein knock down (Figure 4B+C). This effect was specific for HIF1A as the RNA levels of the house-keeping gene GAPDH were unaltered in injected cells (Supplementary Figure S6). These results show that similarly high numbers of LNA-ASO molecules (∼10^5^) are required for efficient knockdown of target RNA and protein.

### Nuclear accumulation and limited diffusion of LNA-ASO within the nucleus

#### Rapid nuclear accumulation of microinjected LNA

**Figure 5.**
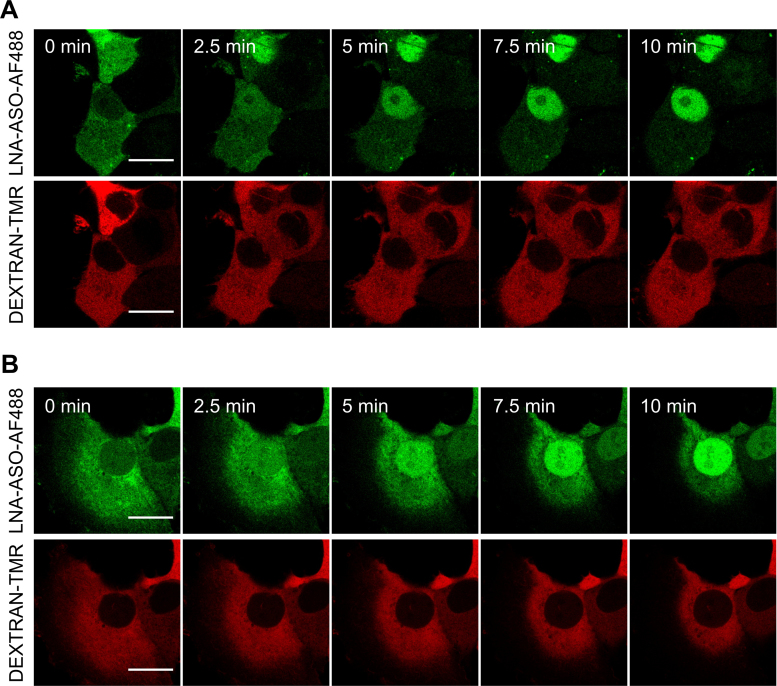
Rapid nuclear accumulation of LNA-ASOs after microinjection. MCF-7 cells were co-injected with 5 μM TMR-dextran (70 kDa) as tracer + 5 μM MALAT1 LNA-AF488 (LNA-ASO-AF488). Directly after injection confocal time lapse imaging was started. LNA rapidly accumulates in the nucleus whereas the tracer remains in the cytosol. Scale bar: 20 μm (**A**). To assess the influence of active transport on nuclear accumulation, cellular ATP pools were depleted before injection. Cells were kept under starvation conditions over night and incubated with 6 mM deoxyglucose and 10 mM sodium azide for 45 min prior to injection. Scale bar: 20 μm (**B**).

Having found that a high number of ∼10^5^ LNA-ASO molecules needed to be injected into the cytosol for efficient target knock down, we applied fluorescence microscopy to visualize how the bulk of injected LNA-ASO became distributed inside cells following microinjection (Figure 5A). To exclude the possibility that the integrity of the nuclear membrane was affected by microinjection, we co-injected a high molecular weight TMR-labelled dextran (70 kDa) into the cytosol, which cannot diffuse freely through nuclear pores. Directly after injection, both LNA-ASO and dextran were detected in the cytosol. While the high molecular weight dextran did not passage through the nuclear membrane, the smaller LNA-ASO was rapidly distributed throughout the whole cell. Within less than five minutes, we observed a strong accumulation of LNA-ASO inside the nucleus. Similar data were obtained for another LNA-ASO compound carrying a different fluorophore (Supplementary Figure S7). Depletion of the intracellular ATP pool by treatment with 6 mM deoxyglucose and 10 mM sodium azide did not significantly affect nuclear translocation of LNA-ASO (Figure 5B), indicating that the nuclear accumulation did not require active transport. Given that oligonucleotides are small enough to pass through nuclear pore complexes, LNA-ASO s can rapidly translocate into the nucleus via passive diffusion, which leads to an even cellular distribution, but does not explain nuclear accumulation. Consistently, a freely diffusible tracer (small molecular weight dextran at 10 kDa) co-injected together with LNA-ASO, was found evenly distributed throughout the cell with no visible nuclear accumulation (Supplementary Figure S7). The finding that LNA-ASOs rapidly accumulated inside the nucleus following passive diffusion indicates that they must be retained inside the nucleus by binding to nuclear components, according to the diffuse and bind model (47).

#### Fluorescence recovery after photobleaching

**Figure 6.**
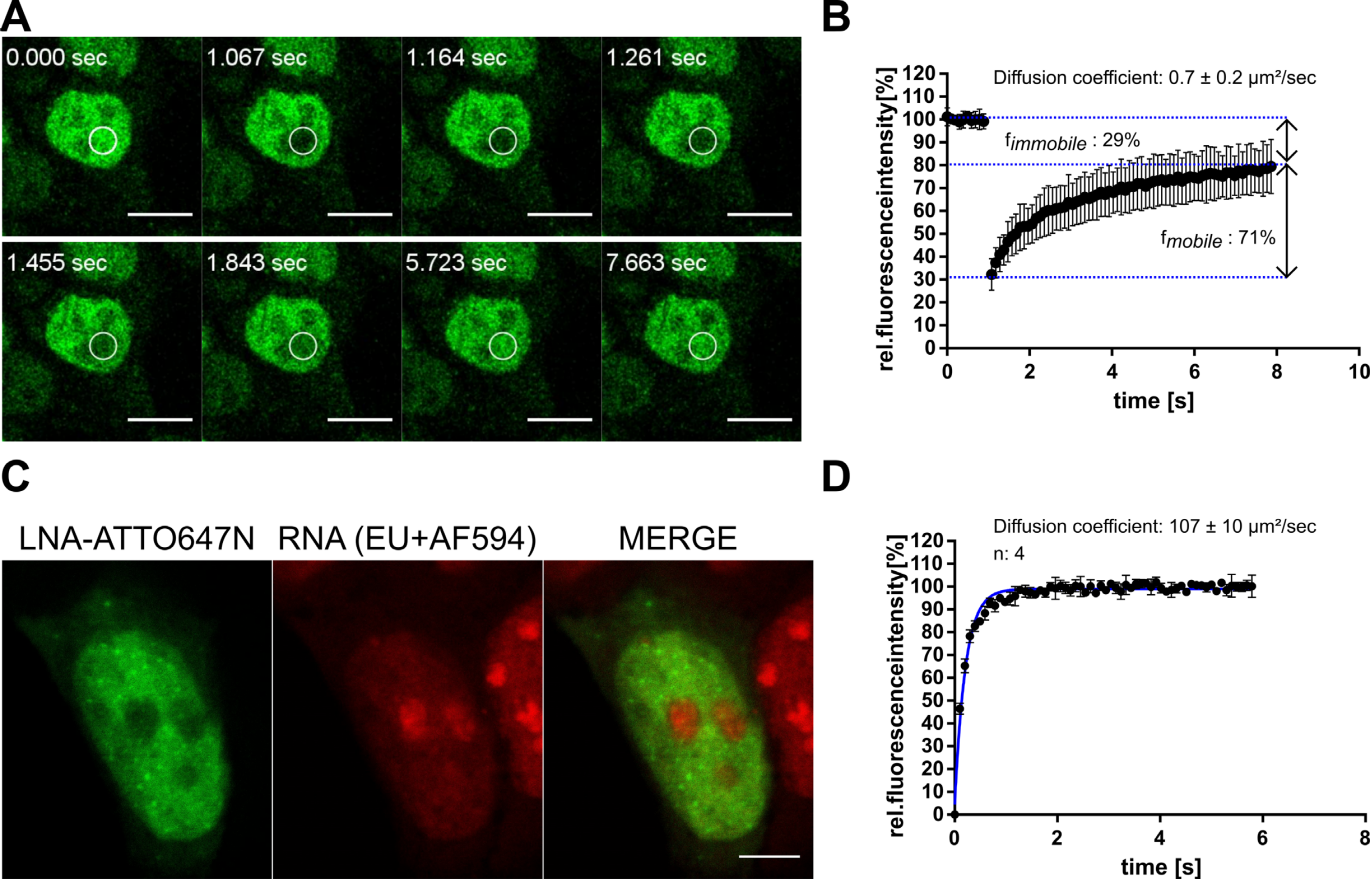
Restricted diffusion of LNA-ASOs inside the nucleus. Fluorescence recovery after photobleaching (FRAP) experiments were performed in microinjected cells using FAM-HIF1A-LNA-ASOs. MCF-7 cells were injected with a solution of 20 μM FAM-LNA-ASOs and incubated for 30 min. Diffusion of the labelled oligonucleotides was assessed by photobleaching a defined region of interest and monitoring the recovery of fluorescence signal within the photobleached area. Scale bars: 10 μm (**A**). The time trace of fluorescence intensity within the photobleached area was recorded, normalized and fit to an exponential model (see Material and Methods). Mean diffusion coefficient ± SD was calculated from the half-life time of fluorescence recovery. Three independent experiments were performed and 30 cells were analyzed in total (**B**). Nucleoli were identified via incubation with 1 mM EU for 1 h, which is incorporated into freshly synthesized RNA. EU-treated cells were injected with a solution of 5 μM LNA-ATTO647N (LNA-ASO-ATTO647). Cells were fixed 20 min after injection and incorporated EU was detected via Click-reaction with AF594-N_3_. Scale bar: 5 μm (**C**). Diffusion of FAM-HIF1A-LNA-ASOs was assessed in aqueous solution performing FRAP experiments under similar conditions as in the nuclear environment. To account for faster recovery kinetics, the radius of the bleached area was increased (**D**).

As a result of binding to nuclear components, nuclear LNA-ASO may exhibit limited diffusion. To investigate whether LNA oligonucleotides can diffuse freely or are bound or compartmentalized inside the nucleus we analyzed LNA-ASO mobility within the nucleus via fluorescence recovery after photobleaching (FRAP) (48). Fluorescent LNA-ASO molecules were delivered to the cytoplasm via microinjection and cells were incubated for 20 min to allow for complete nuclear accumulation of the LNA-ASO. A circular region within the nucleus with 4 μm diameter size was photobleached using a high intensity laser pulse and the recovery of the fluorescence signal in the bleached area was recorded over time (Figure 6A). Analysis of the recovery curve provides information about the apparent diffusion rate of the labelled molecule. As the nuclear architecture is characterized by different compartments, obtained diffusion rates represent an average value originating from different microenvironments. We observed that LNA oligonucleotides accumulated within nuclear foci and were absent in nucleoli. These sites of ribosomal RNA synthesis accounting for 80% of cellular RNA were visualized by incubation with 5-ethynyl uridine (EU) which is incorporated into freshly synthesized RNA (Figure 6C). Therefore, nucleoli were excluded from the analysis. The effective diffusion coefficient of labelled LNA-ASO molecules inside the nucleus was determined from the half-time of the recovery curve as 0.7 ± 0.2 μm²/s (mean ± SD) (Figure 6B). The fluorescence signal did not completely recover to pre-bleached levels indicating a fraction of approximately 29% immobile molecules. In contrast, complete recovery was observed in aqueous solution where the free diffusion of LNA-ASO is characterized by a diffusion coefficient of 107 ± 10 μm²/s (mean ± SD) which is by two orders of magnitude higher compared to the nuclear environment (Figure 6D). The dramatically restricted nuclear diffusion of LNA-ASO even when injected as large excess is a strong indication for substantial binding to macromolecular complexes and consistent with its stable accumulation within the nucleus.

### Knock down of LNA-ASO delivered by *gymnosis*

**Figure 7.**
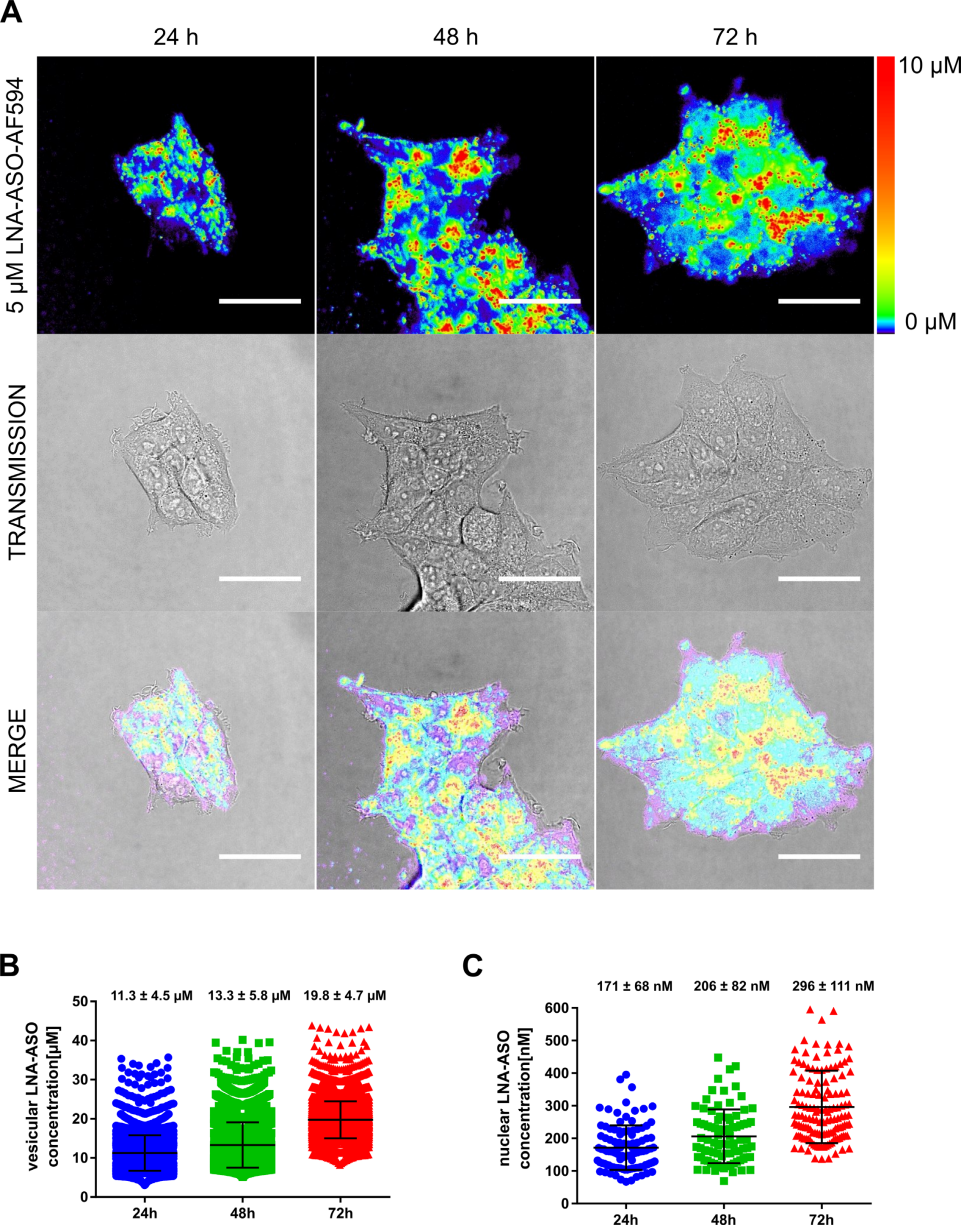
Detection of nuclear LNA-ASOs after gymnotic delivery. MCF-7 cells were incubated with 5 μM HIF1A LNA-AF594 (LNA-AF594 oligonucleotides) for 72 h. Live cell imaging was carried out on a confocal microscope set to photon counting mode. Using a standard curve, photons counts were translated into concentrations and presented as false-color heat map. A logarithmic color scale was applied to visualize differences in the low concentration range. Scale bar: 40 μm (**A**). Vesicular LNA-ASO concentrations were calculated using automated image segmentation (**B**). Nuclei were identified manually using transmission images. LNA-ASO concentrations were measured from average gray values in nuclear areas free of signal contamination originating from vesicles (**C**). Bars represent mean values ± SD. Quantitative analysis of three independent experiments.

Our microinjection studies showed LNA-ASO numbers in the range of 10^5^ molecules need to be delivered into the cytosol to achieve knock down of the target genes. However, as biomedical applications of LNA-ASO cannot rely on microinjection techniques, we assessed the amount of LNA-ASO reaching the nucleus following gymnotic delivery, i.e. uptake of single-stranded oligonucleotides by living cells without the use of any transfection reagents (10). MCF-7 cells were incubated in cell culture medium containing 5 μM fluorescently labelled LNA-ASO for 24, 48 and 72 h. At this extracellular concentration, HIF1A RNA levels were decreased by 50% as determined by qPCR (Supplementary Figure S8). Comparing unlabelled and labelled LNA-ASO side by side, we did not observe any difference in potency, indicating that the fluorescent label does not interfere with uptake, routing and mechanism of knock down (Supplementary Figure S8). As fixation of cells has been observed to induce artificial nuclear localization (49), we analyzed living cells by confocal imaging in photon counting mode allowing the generation of quantitative false-colour heat maps of intracellular LNA-ASO concentrations. As expected, the majority of intracellular LNA-ASO was contained in the endo-lysosomal compartment (Figure 7A). Using image segmentation and calibration of grey values, we calculated the average vesicular concentrations as 11.3 ± 4.5, 13.3 ± 5.8 and 19.8 ± 4.7 μM LNA-ASO after 24, 48 and 72 h incubation, respectively (mean ± SD) (Figure 7B). From image segmentation, we determined an average vesicular volume of 1.014 ± 0.018 fl (mean ± SEM, n: 5061 vesicles). Therefore, a single vesicle contains about 12 000 LNA-ASO molecules after 72 h incubation. With 52 ± 9 vesicles (mean ± SD) per cell (as determined from maximum projections of 3D images), we estimate the number of LNA-ASO molecules within the endosomal compartment to approximately 620,000/cell.

In addition to the bright vesicular signal, the logarithmically colour-coded heat map revealed low amounts of nuclear LNA. By manually selecting nuclear regions of interests free of vesicular signals we determined the nuclear LNA-ASO concentrations as 171 ± 68, 206 ± 82 and 296 ± 111 nM after 24, 48 and 72 h, respectively (mean ± SD) (Figure 7C). Assuming a nuclear volume of 1180 fl (50), this corresponds to a total number of 210 000 ± 80 000 (mean ± SD) LNA-ASO molecules in the nucleus, in good agreement with the number of molecules (∼10^5^) required for functional knock down determined by microinjection experiments.

## DISCUSSION

In this study, we determined the absolute number of LNA-ASO molecules required for knock down of two target genes detected either at RNA or protein level. Intracellular LNA-ASO levels were quantified by fluorescence microscopy, comparing gymnotic uptake with direct cytosolic delivery via microinjection. In parallel, we followed the subcellular distribution of fluorescently labelled LNA-ASOs and measured the mobility/diffusion constant of LNA-ASOs within the nucleus of living cells.

### The amount of LNA required for knock down is substantially higher than cellular RNA levels

Given the many variables determining LNA-ASO potency *in vitro*, microinjection of LNA oligonucleotides into the cells allowed us to focus on their activity at the subcellular site of action, leaving out variables such as stability and protein binding in medium, and more importantly uptake into cells via endocytosis and endosomal escape (51). Delivery of a defined amount of unlabelled LNA-ASOs into the cytosol and subsequent single cell knock down analysis revealed that ∼10^5^ LNA-ASO molecules are required for efficient target knock down. The absolute number of LNA-ASO molecules was established both at RNA and protein level using the noncoding RNA MALAT1 and the nuclear protein HIF1A as model targets.

Calculations on LNA-ASO molecules required for target gene suppression have been based on copy numbers of RNA substrates, cellular RNaseH1 levels and cleavage rate of the enzyme. Compared to relatively high cellular levels of RNaseH1 (1,000 copies/cell) and a fast processing rate of 7.1 cleavages/sec per RNase H1 molecule (51,52), the amounts of catalytically active LNA-ASOs determined here for efficient target knock down were higher than what would be expected from these numbers. The median copy number of a typical mRNA is 17 mRNAs per cell with a transcription rate of 2 mRNAs/h and a median half-life of 9 h (44). In this regard, the non-coding RNA used in this study, MALAT1, is relatively abundant with a copy number of 2,500 RNAs per cell (43). For the second target, HIF1A, we found comparable mRNA copy numbers in MCF-7 cells (as determined by qPCR) consistent with high protein turnover enabling fast adaption to changing oxygen conditions. Efficient down regulation of comparably abundant targets by RNA interference was reported by applying <2000 siRNA molecules per cell using lipid nanoparticle transfer (42,53). Surprisingly, the number of LNA-ASO molecules required for knock down is more than an order of magnitude higher than the copy number of both investigated target RNAs, indicating that only a small fraction might be available for RNase H1 mediated RNA knock down.

### A large fraction of LNA-ASO is bound to nuclear components and might not be available for RNase H1 mediated target degradation

Investigating the underlying mechanism, we followed the distribution of fluorescently labelled LNA-ASOs inside living cells following microinjection. While considerable attention has been given to the endosomal trafficking of antisense oligonucleotides, little has been known about LNA-ASO trafficking to and especially within the nucleus (28,29). Our investigations on LNA-ASO trafficking after delivery by microinjection revealed that LNA oligonucleotides rapidly redistributed from the cytosol to the nucleus, suggesting that nuclear transport is not a bottleneck for subcellular LNA-ASO delivery. This observation is consistent with earlier reports on nuclear accumulation of microinjected single stranded oligonucleotides which has been observed both with fluorescently tagged and BrdUrd-modified oligomers, whose nuclear distribution was monitored by indirect immunofluorescence with BrdUrd-specific antibodies (54,55). In these studies, fluorescence microscopy data showed that the bulk of single-stranded antisense-oligonucleotides injected into the cytosol almost quantitatively accumulated inside the nucleus. However, small amounts of cytosolic LNA-ASOs might be missed due to the detection limit (∼1000 molecules). Cytosolic antisense effects through steric blocking or trapping of LNA-ASOs by binding to cytosolic proteins should be considered when low numbers of oligonucleotides are delivered to the cytosol, i.e. after endosomal escape. The question how antisense oligonucleotides are translocated and enriched inside the nucleus is still under debate. For oligonucleotides modified with a phosphorothioate backbone, rapid nuclear accumulation has been attributed to active nucleocytoplasmic shuttling whereas data obtained from phosphodiester oligonucleotides argue for passive transport by diffusion (54,56,57). Following depletion of the intracellular ATP pool before microinjection, we found that LNA-ASO transport to the nucleus was energy independent and mediated by diffusion through the nuclear pore complexes, either in a free form or bound to protein complexes. We cannot exclude that active nuclear transport might play a role in the natural situation after unassisted endocytic uptake. However, the observation of nuclear LNA-ASO accumulation after microinjection together with passive nuclear transport via diffusion clearly indicates that LNA oligonucleotides must be retained inside the nucleus by binding to nuclear components.

FRAP experiments in the nucleus of living cells revealed that the diffusion of LNA-ASO is highly constrained, indicating binding to less mobile macromolecular structures. This is in contrast to diffusion measurements reported by Politz *et al*. (48) using fluorescein-labelled oligo(dA) and (dT) (43-mers) for which a significant fraction of freely diffusing oligomers showed nuclear diffusion rates similar to those measured in aqueous solution. Although intracellular ‘fluid-phase’ viscosity, defined as the microviscosity sensed by a small probe in the absence of molecular interactions, is only 1.2–1.4 times greater than the viscosity of water (58), translational diffusion of macromolecules on a larger scale is hindered by molecular crowding. Unreactive macromolecules such as dextrans show nuclear diffusion rates 3- to 5-fold slower than in water while binding to immobile obstacles further constrains the mobility of nuclear solvents by orders of magnitude (59–61). Thus, our finding that nuclear LNA-ASO diffusion rates are by two orders of magnitude reduced clearly indicates binding to immobile nuclear components.

The high number of LNA-ASO molecules required for knock down together with the strong binding to nuclear components suggests that a large fraction of LNA oligonucleotides might be sequestered and therefore not be available for RNase H1-mediated target knock down. This notion is further supported by our competition experiments with unrelated LNA. We found that co-injection of excess of unrelated LNA-ASO increased the potency of the oligonucleotides, presumably by saturating non-target RNA binding sites. This is in line with competition experiments *in vivo*: co-administration of ‘nonsense’ oligonucleotides significantly improved target mRNA knockdown in liver suggesting that the ‘nonsense’ oligonucleotides compete for uptake into an apparent negative or non-productive sink (62). Our results indicate that binding to nuclear components might represent at least part of this sink and that the activity of antisense oligonucleotides is influenced by competition between RNA target and nuclear protein binding. Indeed, a number of nuclear and cytosolic proteins have been identified which can interact with the duplex formed between LNA-ASO and target RNA and compete with RNase H1 for binding (23,63,64). While interaction with nucleic-acid binding proteins has been found for all phosphorothioated oligonucleotides, chemical modifications at the 2′ position of the ribose ring seem to strongly influence binding to proteins (23). LNA oligonucleotides have a strong tendency to bind Hsp90 which is reported to enhance their activity (65). As a possible mode of action, chaperone proteins might modulate antisense activity by melting potential intramolecular structures or simply by preventing binding to inhibitory proteins. Additionally, interaction with nuclear or cytosolic proteins can enhance or reduce the activity of antisense oligonucleotides by influencing their subcellular localization. Indeed, we observed the formation of LNA-ASO nuclear foci upon microinjection. Interaction with the paraspeckle protein P54nrb was reported to negatively affect antisense activity of oligonucleotides via formation of paraspeckle-like structures (66). In contrast, interaction with TCP1 in distinct nuclear structures of 0.15–2.0 μm in diameter, termed phosphorothioate bodies (PS-bodies), was related to increased knock down activity (67). Given that interaction with different cellular components seems to strongly influence LNA-ASO activity in both ways, screening for beneficial binding properties will make a valuable contribution to increase the potency of therapeutic LNA oligonucleotides.

### Following gymnotic uptake, a substantial amount of LNA-ASO can escape from endosomes and reach the nucleus

In a last set of experiments, we compared our results from microinjection experiments with gymnotic delivery of LNA-ASO and assessed the amount of LNA-ASO reaching the nucleus. Quantification of nuclear LNA-ASO levels in living cells was achieved for the first time by applying high sensitivity fluorescence imaging. Since the fluorescence labelling had no effect on LNA-ASO potency, we expect that our findings are transferable to unlabelled oligonucleotides. This assumption is supported by the fact that the number of labelled LNA-ASO molecules delivered to the nucleus via free uptake is in good in line with the copy number required for knock down as determined by microinjection experiments using unlabelled LNA.

When applying low micromolar extracellular LNA-ASO concentrations, copy numbers in the range of 10^5^ LNA-ASO molecules were detected in the nucleus. We found that approximately a quarter of total cellular LNA-ASO can be found in the nucleus, indicating that endosomal escape is not a rare event for LNA-ASO. This is of significance for therapeutic applications since endosomal escape rates for siRNA and other antisense oligonucleotides have been estimated to 0.01%, thereby representing the major bottleneck for antisense oligonucleotide delivery (6). Having found that the presence of high LNA-ASO copy numbers in the nucleus is required for knock down activity, efficient endosomal escape and nuclear accumulation remain a critical factor determining the potency of LNA oligonucleotides. In addition, dynamic processes within the nucleus seem to have a strong influence on LNA-ASO activity and require further investigations in order to increase the potency of LNA oligonucleotides for therapeutic applications. This could mean that a loading dose may be applied first, followed by maintenance doses. In this situation, the first dose might partially saturate unspecific binding sites.

Another major aspect for improving LNA-ASO delivery includes strategies for efficient internalization into the endosomal compartment, given that endosomal LNA-ASO concentrations were found moderately enhanced by a factor of 4 compared to the extracellular space following gymnotic uptake as shown in this study. Delivery approaches that target cell surface receptors, such as GalNAc- or antibody-conjugation, provide more efficient internalization kinetics, i.e. stronger endosomal enrichment of LNA oligonucleotides in shorter time (6,26). In conclusion, our data provide a functional and quantitative basis for the development of new strategies to efficiently deliver LNA oligonucleotides to their desired cellular and subcellular target sites.

## Supplementary Material

Supplementary Data
