# Supplementary material for: Quantitative fluorescence imaging determines the absolute number of locked nucleic acid oligonucleotides needed for suppression of target gene expression
Source: Nucleic Acids Res. 2018 Nov 20;47(2):953–69. doi: 10.1093/nar/gky1158 (PMC6344898; doi:10.1093/nar/gky1158)
Supplement: Supplementary Data [file gky1158_supplemental_files.pdf]

Supplementary Information

Quantitative fluorescence imaging determines the absolute number of locked nucleic acid oligonucleotides needed for suppression of target gene expression

Buntz Annette[1], Killian Tobias[1], Schmid Daniela[1], Seul Heike[1], Brinkmann Ulrich[1], Ravn Jacob[2], Lindholm Marie[2], Knoetgen Hendrik[3], Haucke Volker[4], Mundigl Olaf[1]*

[1] Roche Innovation Center Munich, Roche Pharma Research and Early Development, Penzberg, 82377, Germany

[2] Roche Innovation Center Copenhagen, Roche Pharma Research and Early Development, Hørsholm, 2970, Denmark

[3] Roche Innovation Center Basel, Roche Pharma Research and Early Development, Basel, 4070, Switzerland

[4] Department of Molecular Pharmacology and Cell Biology, Leibniz-Forschungsinstitut für Molekulare Pharmakologie, Berlin, 13125, Germany

* To whom correspondence should be addressed. Tel: +498856602854; Fax: +498856602854; Email: olaf.mundigl@roche.com

A

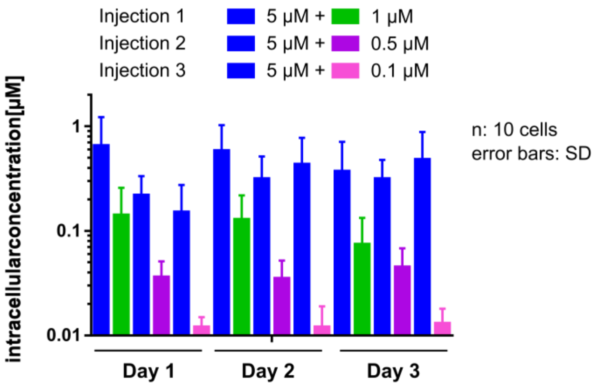

	Injection 1		Injection 2		Injection 3	
**Injection concentration**	**5 µM**	**1 µM**	**5 µM**	**0.5 µM**	**5 µM**	**0.1 µM**
Intracellular concentration day 1	0.650 ± 0.574 µM	0.141 ± 0.117 µM	0.218 ± 0.116 µM	0.036 ± 0.015 µM	0.151 ± 0.124 µM	0.012 ± 0.003 µM
Intracellular concentration day 2	0.581 ± 0.442 µM	0.128 ± 0.090 µM	0.314 ± 0.200 µM	0.035 ± 0.017 µM	0.431 ± 0.344 µM	0.012 ± 0.007 µM
Intracellular concentration day 3	0.370 ± 0.340 µM	0.074 ± 0.059 µM	0.314 ± 0.164 µM	0.045 ± 0.023 µM	0.480 ± 0.403 µM	0.013 ± 0.005 µM
**Intracellular concentration mean ± SD**	**0.534 ± 0.146 µM**	**0.114 ± 0.036 µM**	**0.282 ± 0.055 µM**	**0.039 ± 0.006 µM**	**0.354 ± 0.178 µM**	**0.012 ± 0.001 µM**

B

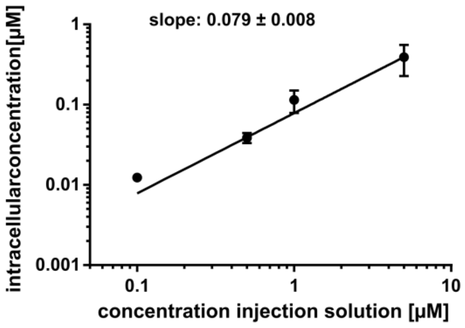

Injection solution	Intracellular concentration (± SD)
5 µM	390 ± 163 nM
1 µM	114 ± 36 nM
0.5 µM	39 ± 6 nM
0.1 µM	12 ± 1 nM

Supp. Figure 1: Microinjection calibration via photon counting imaging. For calibration, intracellular tracer concentrations were measured after delivery by microinjection. MCF-7 cells were injected with solutions containing 5 µM dextran-AF647 (10 kDa) + 1 / 0.5 / 0.1 µM dextran-AF488 (10 kDa). The intracellular concentration of each molecular species was determined by fluorescence imaging in photon counting mode. The number of photons detected at every pixel was translated into concentrations using a standard curve. Bars represent cellular mean concentrations ± SD. Ten cells were analyzed per condition and experiments were repeated on three days (**A**). Intracellular LNA oligonucleotide concentrations were plotted against injection concentration yielding a calibration curve for microinjection experiments under the given injection conditions. Average intracellular concentrations were calculated from mean values of three independent experiments (**B**).

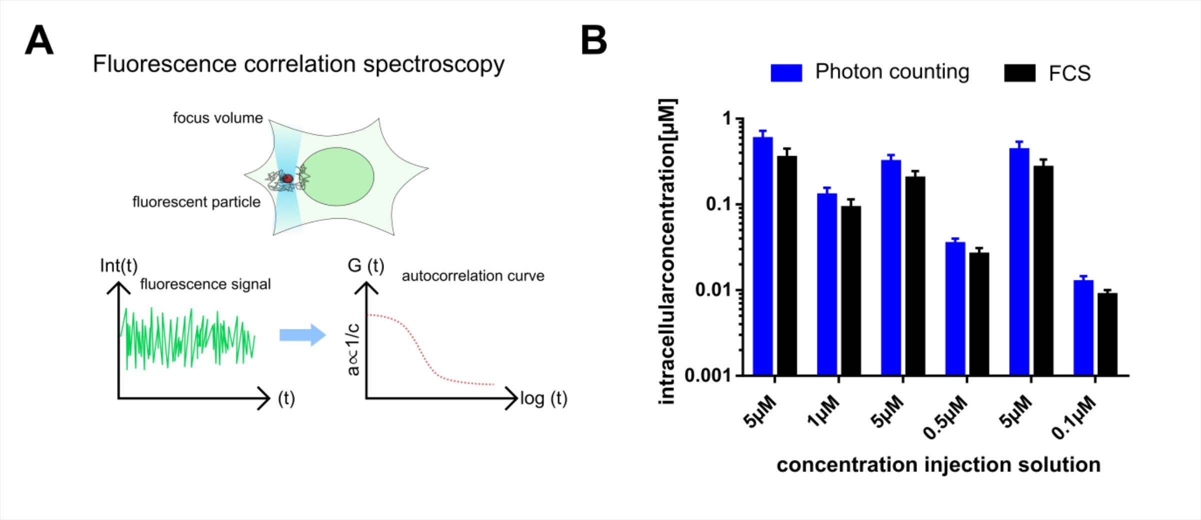

Supp. Figure 2: Comparison photon counting imaging and FCS. Quantification of intracellular LNA oligonucleotide concentrations were confirmed by fluorescence correlation spectroscopy (FCS). MCF7- cells were injected with 5 µM dextran-AF647 + 1 / 0.5 / 0.1 µM dextran-AF488, first analyzed by photon counting imaging, and then subjected to concentration measurements via FCS. Intensity fluctuations of AF488 and AF647 signals within the confocal volume were recorded over time and the amplitude of the resulting autocorrelation curve was translated into cellular concentrations (**A**). Minor photobleaching during photon counting imaging explains the systematically lower concentrations obtained from subsequent FCS measurements. Three point measurements were performed per cell and ten cells were analyzed per injection condition (**B**).

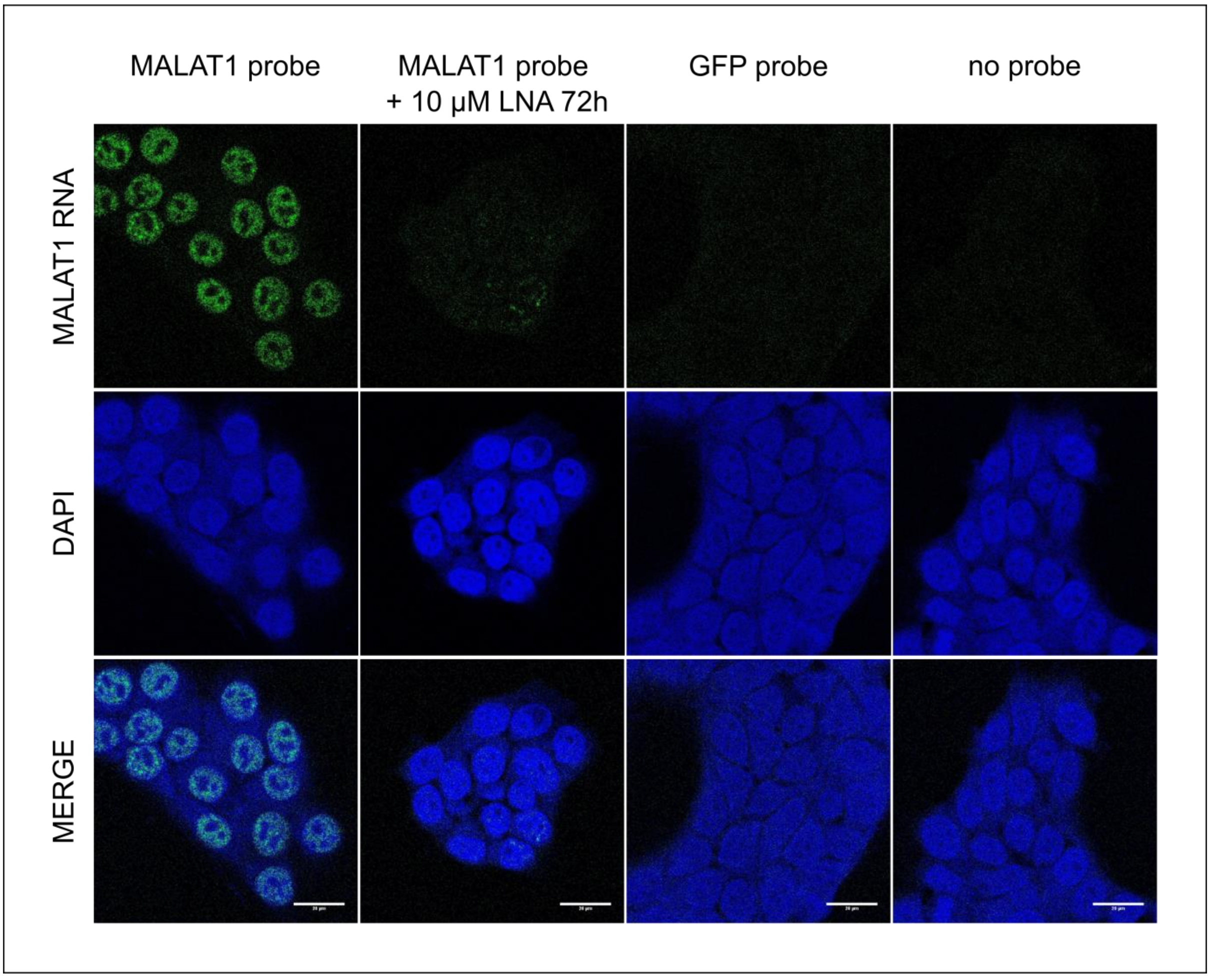

Supp. Figure 3: Specific detection of MALAT1 RNA by FISH. MALAT1 RNA was detected in intact cells using fluorescence in-situ hybridization. MCF-7 cells were fixed with 4% PFA and permeabilized with 70% EtOH at 4°C. Fluorescence in-situ hybridization was carried out overnight using a mixture of fluorescently labeled probes which specifically bind to different complementary regions on the MALAT1 RNA sequence (first panel). Nuclei were stained with DAPI. As control, MALAT1 RNA was knocked down by incubation with 10 µM MALAT1 LNA-ASOs for 72 h (second panel). Treatment with a GFP probe which has no complementary RNA sequence in MCF-7 cells or incubation hybridization without probe did not produce any fluorescence signal (third and fourth panel). Scale bar: 20 µm.

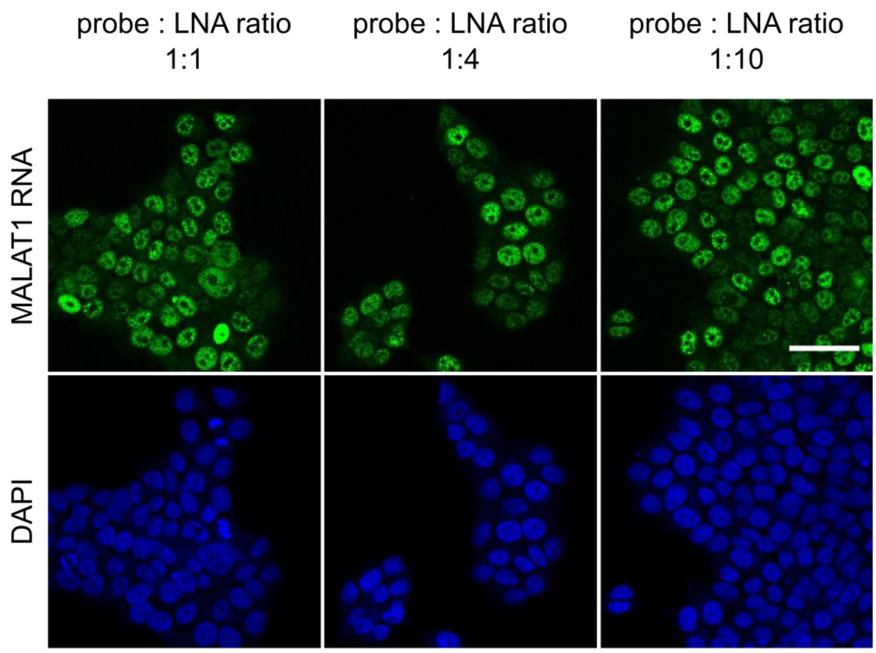

Supp. Figure 4: LNA-ASOs do not interfere with in situ hybridization methodology. The presence of MALAT1 targeting LNA-ASOs does not interfere with in-situ hybridization. MCF-7 cells were fixed with 4% PFA, permeabilized with 70% EtOH at 4° C and incubated with fluorescently labelled MALAT1 probes for hybridization together with different concentrations of MALAT1 targeting LNA-ASOs. Scale bar: 50 µm.

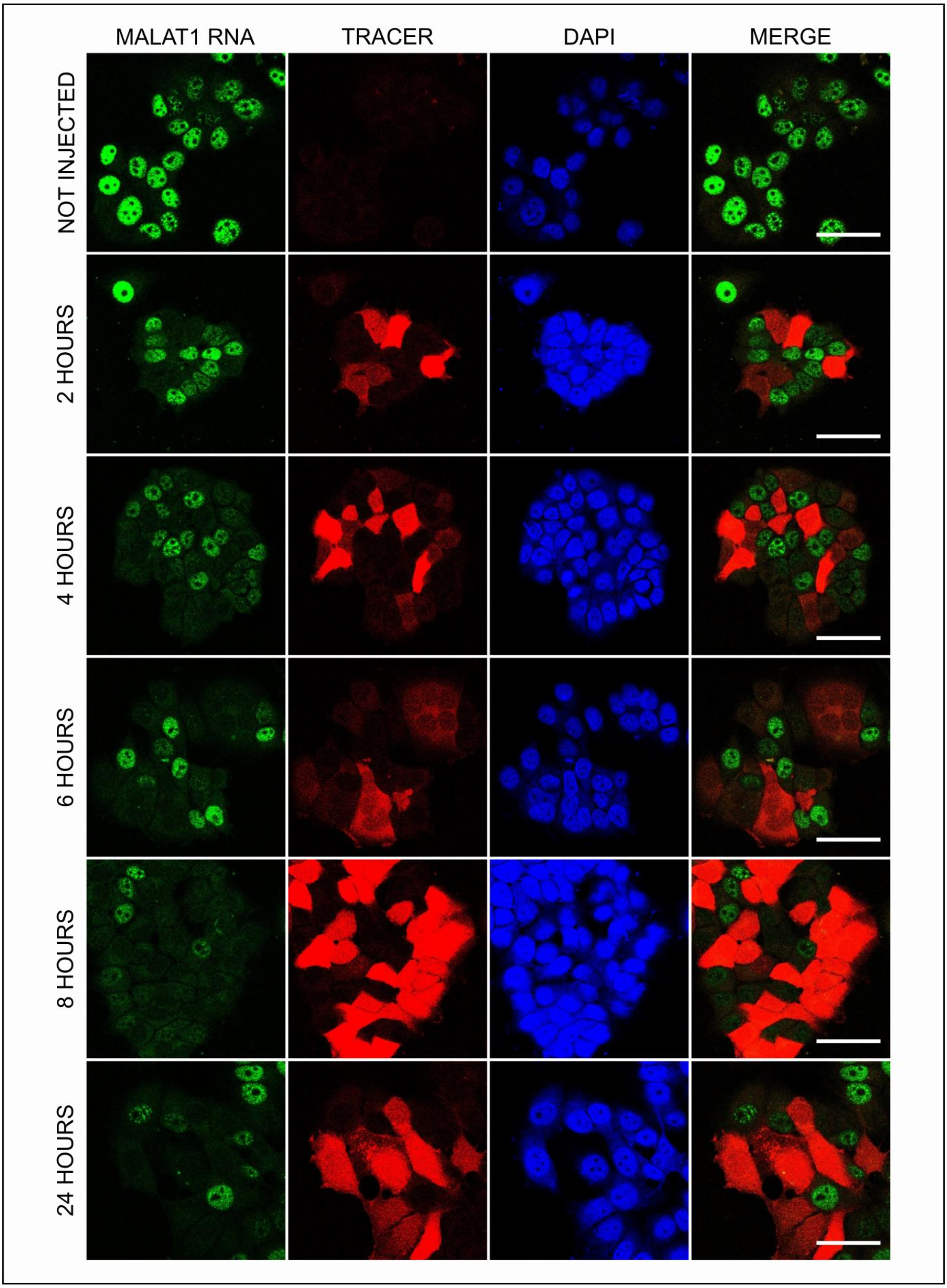

Supp. Figure 5: Time course MALAT1 KD. Time-course of MALAT1 knock down following microinjection of LNA-ASOs. MCF-7 cells were microinjected with a solution containing 10 µM dextran-AF488 as tracer + 1 µM unlabeled MALAT1 LNA-ASOs and incubated as indicated prior to fixation with 4% PFA. Following

permeabilization with 70% EtOH, MALAT1 RNA was detected in intact cells via fluorescence in situ hybridization. Nuclei were stained by DAPI and cells were analyzed using confocal fluorescence microscopy. Scale bar: 50 µm.

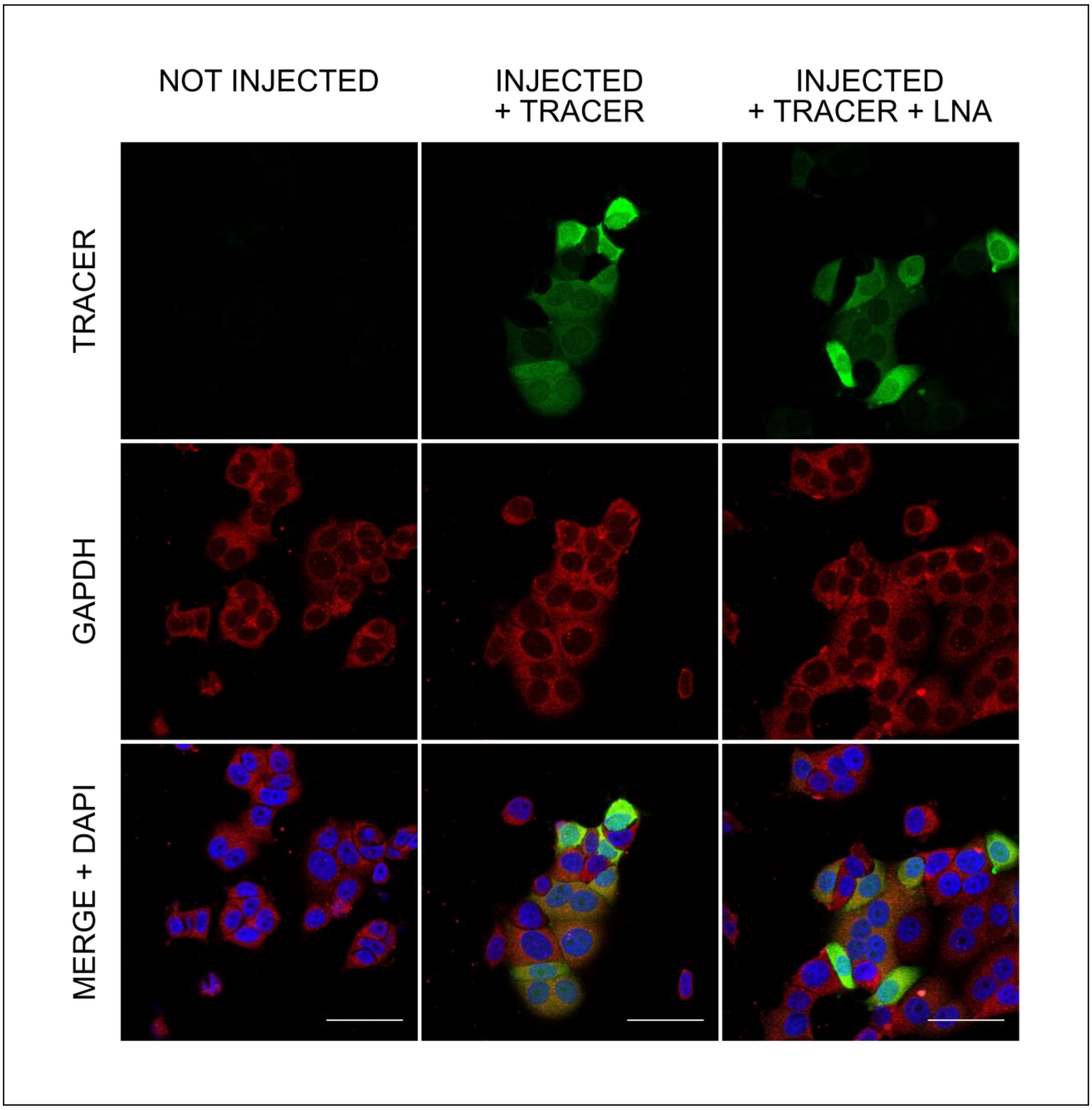

Supp. Figure 6: Control GAPDH levels in injected cells. GAPDH levels are not affected by microinjection. MCF-7 cells were either left untreated, injected with 10 µM dextran-AF488 as tracer or co-injected with tracer and 1 µM unlabeled HIF1A LNA-ASOs. Cells were incubated with 100 µM deferoxamine for 48 h and fixed using 4%PFA. GAPDH RNA was detected in permeabilized cells via fluorescence in situ hybridization with a fluorescent RNA probe. Scale bar: 50 µm.

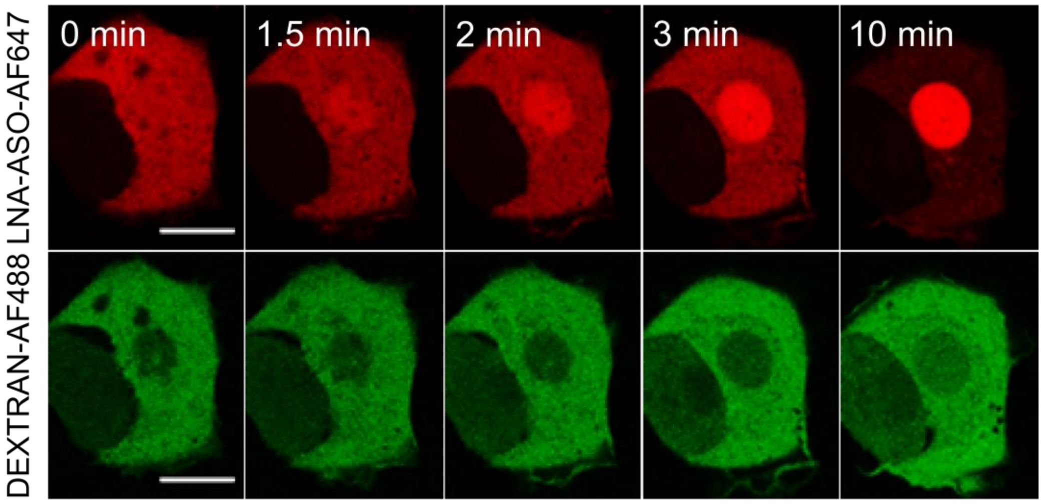

Supp. Figure 7: Nuclear accumulation of HIF1A LNA-ASO-AF647. MCF-7 cells were co-injected with a solution of 5 µM dextran-AF488 (10 kDa) as tracer + 5 µM HIF1A LNA-ASO-AF647. Directly after microinjection confocal time lapse imaging was started. LNA-ASOs rapidly accumulated in the nucleus whereas the tracer remained evenly distributed throughout the cell. Scale bar: 20 µm.

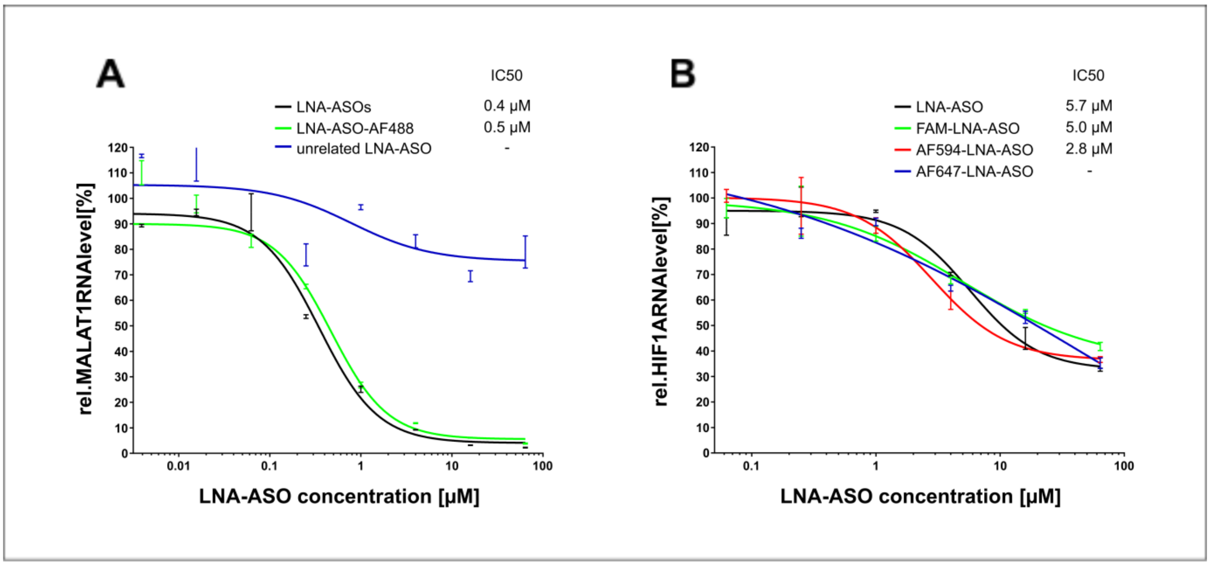

Supp. Figure 8: Influence of fluorescence labeling on knock down efficiency of LNA-ASOs. Relative MALAT1 (**A**) and HIF1A (**B**) RNA levels were measured by qPCR of MCF-7 cell lysates after incubation with indicated LNA-ASOs for 72 h (gymnotic uptake). Bars represent mean values ± SEM of technical duplicates.
